# Identification of a novel macrophage-related prognostic signature in colorectal cancer

**DOI:** 10.1038/s41598-024-53207-9

**Published:** 2024-02-02

**Authors:** Dongfa Lin, Tingjin Zheng, Shangyuan Huang, Rui Liu, Shuwen Guan, Zhishan Zhang

**Affiliations:** 1 Key Laboratory for Molecular Enzymology and Engineering, The Ministry of Education, Jilin University, School of Life Sciences, Changchun, 130012 China; 2 Engineering Laboratory for AIDS Vaccine, Jilin University, Changchun 130012, China; 3 School of Life Sciences, Jilin University, Changchun 130012, China; 4 Department of Clinical Laboratory, Quanzhou First Hospital Affiliated to Fujian Medical University, No. 248 East Street, Quanzhou, 362000 Fujian China; 5 Laboratory of Molecular Neurobiology, Sheng Yushou center of Cell Biology and Immunology, Department of Genetics and Developmental Biology, School of Life Sciences and Biotechnology, Shanghai Jiao Tong University, 800 Dongchuan Rd., Shanghai, 200240 China

**Keywords:** Cancer, Gastroenterology

## Abstract

Colorectal cancer (CRC) is one of the most prevalent and deadliest illnesses all around the world. Growing proofs demonstrate that tumor-associated macrophages (TAMs) are of critical importance in CRC pathogenesis, but their mechanisms remain yet unknown. The current research was designed to recognize underlying biomarkers associated with TAMs in CRC. We screened macrophage-related gene modules through WGCNA, selected hub genes utilizing the LASSO algorithm and COX regression, and established a model. External validation was performed by expression analysis using datasets GSE14333, GSE74602, and GSE87211. After validating the bioinformatics results using real-time quantitative reverse transcription PCR, we identified SPP1, C5AR1, MMP3, TIMP1, ADAM8 as potential biomarkers associated with macrophages in CRC.

## Introduction

Colorectal cancer (CRC) is a malignant tumor that develops from the colon or rectum and is one of the most prevalent malignant tumors among the globe. The International Agency for Research on Cancer statistics showed 19.3 million cases of cancer diagnosed in 2020, of which CRC accounted for 10%, making it the second most common cause of cancer death following lung cancer. Those statistics have shown that CRC has become a major public hygiene problem. The European project ColoMARK, which aims at identifying new biomarkers of CRC in liquid biopsy samples, has disbursed funding for detection of potential biomarkers for risk prediction of CRC, demonstrating that it’s essential to prevent CRC through early diagnosis by testing biomarkers. Major risk factors of CRC include obesity, diet, smoking, and physical inactivity. Dietary factors including processed meat, red meat, and alcohol, raise risk of CRC^1^. Some genetic syndromes are also related to a high incidence of CRC. Mutations in some of the genes are already accepted to be relevant to CRC^2,3^, while there might still exist epigenetic factors and other underlying mechanisms in the development of CRC^4^. Further research is needed on the pathogenesis of CRC and its related biomarkers.

Over the years, a growing number of reports have indicated the important role of tumor microenvironment (TME)^5,6^ in tumor progression. TME refers to the environment that surrounds the tumor, encompassing immune cells, fibroblasts, extracellular matrix, surrounding blood vessels, etc. Tumors can release extracellular signals that may affect the microenvironment and alter immune cells, thus facilitating tumor angiogenesis and inducing immune tolerance. Among these immune cells, macrophages are the most important phagocytes in vivo and are widely recognized to have a vital part in the mechanisms of tumor development^6^. The macrophages are divided into two subsets, named classically activated (M1) macrophages and alternatively activated (M2) macrophages. M2 macrophages, unlike M1 macrophages, secrete various anti-inflammatory factors like arginase1, TGF-β, and IL-10. Studies have shown that most of the infiltrated macrophages in tumors are considered to have an M2-like phenotypes^7^, which might secrete cytokines, chemokines and proteases to provide an immunosuppressive environment for tumor angiogenesis and growth. Those macrophages that reside within TME are called tumor-associated macrophages (TAMs). Research has shown that TAMs can be utilized as possible biomarkers for breast cancer diagnosis and emerging therapy^8^. Identifications of those TAMs might be an effective approach in early cancer diagnosis. Hence, in the treatment and prevention of colorectal cancer, increasing the number of M1 macrophages or altering their function may help improve patient outcomes. However, it is essential to note that the role of macrophages in colorectal cancer is highly complex and regulated by various factors. Therefore, when assessing patient prognosis, factors such as disease stage, patient age and gender, comorbidities, among others, should be considered in addition to the presence and quantity of macrophages^9^. These factors collectively influence the prognosis of colorectal cancer patients.

However, there are few systematic studies that elucidate the immune microenvironment characteristics of CRC and the types of immune cells, particularly TAMs. Therefore, our research aimed to identify possible macrophage-related biomarkers in CRC and to study the impact of TAMs on CRC.

## Materials and methods

### Source of data

The details of the data sources can be found in the supplementary files, Tables 1 and S1.

**Table 1:** COADREAD dataset information list.

	TCGA-COADREAD	GSE14333	GSE74602	GSE87211
Platform		GPL570	GPL6104	GPL13497
Species	Homo sapiens	Homo sapiens	Homo sapiens	Homo sapiens
Samples in normal group	51		30	160
Samples in COADREAD group	644	290	30	203
Reference	^45^	^46^	^47^	^48^

*TCGA* the cancer genome atlas, *COADREAD* colon adenocarcinoma/rectum adenocarcinoma esophageal carcinoma.

We employed R package TCGAbiolinks^45^ to download the expression matrix of CRC (colon adenocarcinoma/rectum adenocarcinoma esophageal carcinoma, COADREAD) dataset TCGA-COADREAD from the cancer genome atlas (TCGA, https://portal.gdc.cancer.gov/), eliminated samples missing key clinical information, and obtained 644 CRC samples (cancer group, grouping: COADREAD) and 51 paracancer samples (normal group, grouping: Normal), and they were normalized into Fragments Per Kilobaseper Million (FPKM) format, and UCSC Xena database^49^ (http://genome.ucsc.edu) was utilized to acquire corresponding clinical data. R package limma^13^ was employed to normalize the count sequencing data of TCGA-COADREAD dataset.

We obtained the COADREAD-related datasets GSE14333^46^, GSE74602^47^ and GSE87211^48^ from the GEO database^50^ via R package GEOquery^51^. For GSE14333, Homo Sapiens was selected, and GPL570 [HG-U133_Plus_2] Affymetrix Human Genome U133 Plus 2.0 Array served as data platform. GSE14333 contained microarray gene expression profile data of 290 CRC patient samples. GSE74602 from Homo Sapiens, GPL6104 Illumina humanRef-8 v2.0 expression beadchip, containing microarray gene expression profiles from 30 CRC patient samples and 30 fully matched normal tissue samples adjacent to cancer. GSE87211 from Homo Sapiens, GPL13497 Agilent-026652 Whole Human Genome Microarray 4 × 44 K v2 (Probe Name version), a total of 203 CRC patient samples and 160 partially matched paracancer normal tissue samples were included in the microarray gene expression profile data. All samples were included in this study. The datasets were annotated with the corresponding GPL platform files, and all three GEO datasets were used as validation sets (Table 1).

We collected MRGs from the GeneCards^52^ database, which provides comprehensive information on human genes (https://www.genecards.org/). In the GeneCards database, only MRGs with "Protein Coding" and Relevance score > 5 were retained after searching for "Macrophage" as a keyword, and a total of 576 MRGs were obtained. We obtained 92 MRGs from the references and then combined and de-duplicated them to obtain a total of 637 MRGs (Table S1).

We downloaded somatic mutation data from TCGA-COADREAD dataset from the TCGA website including data such as SNP (single nucleotide polymorphism) and visualized the data using the R package maftools^53^. To analyze copy number variation (CNV) in COADREAD patients, R package TCGAbiolinks was employed to download "Copy Number Variation" data of TCGA-COADREAD dataset and then the data were integrated for GISTIC 2.0 analysis^54^, using default settings for the analysis parameters. We obtained the data of tumor mutation burden (TMB) and microsatellite instability (MSI) of TCGA-COADREAD dataset by downloading from cBioPortal for Cancer Genomics database (https://www.cbioportal.org/)^55^.

### Normalization and merging of datasets

To explore the underlying mechanisms and associated biological characteristics and pathways of differential genes in the cancer and normal groups of COADREAD, we first normalized the datasets TCGA-COADREAD, GSE14333, GSE74602, and GSE87211 using R package limma, and then used R package sva^10^ for the COADREAD datasets GSE14333, GSE74602, GSE87211 by removing batch effects to obtain the combined GEO dataset COADREAD-dataset, and compare the before and after batch effects by distribution box line plots and principal component analysis (PCA) plots.

### Calculation of macrophage scores

The single sample gene set enrichment analysis (ssGSEA)^11^ algorithm enables quantification of the relative abundance of individual genes within a given dataset. We used R packet GSVA^12^ and computed macrophage scores (MS) of all samples in TCGA-COADREAD and GEO datasets by ssGSEA algorithm in accordance with the MRGs expression matrix of all samples in CRC dataset. Then, the expression differences of MS between groups with low and high scores in TCGA-COADREAD and GEO datasets were calculated utilizing Mann–Whitney *U* test, with P < 0.05 considering as statistical significance.

To recognize differentially expressed genes (DEGs) associated with MS grouping, limma package^13^ was employed for analyzing differences in the expression profile of TCGA-COADREAD dataset. DEGs between the MS groups of COADREAD patients were acquired. Screening | logFC|> 0 and P < 0.05 genes as DEGs for further study. Genes were deemed up-regulated differential genes with logFC > 0 and P < 0.05, and down-regulated differential genes with logFC < 0 and P < 0.05.

To obtain macrophage-related DEGs (MRDEGs) associated with COADREAD disease, Firstly, the intersection of MRGs and prognostic genes of CRC was selected, and then the intersection of DEGs in the dataset TCGA-COADREAD was selected with the above genes. R package ggplot2 was employed to generate volcano plots and heatmaps and visualize differential analysis results. In addition, the positions of MRDEGs on human chromosomes were annotated utilizing R-package RCircos.

### Prognostic analysis

Kaplan–Meier (KM) curve analysis is a method of analyzing and inferring patient survival time based on data, studying relationship and degree of outcome with many influencing factors, also known as survival analysis or survival rate analysis. It was proposed by Kaplan and Meier, hence the name Kaplan–Meier method, usually abbreviated as KM method. The KM approach estimates the survival curve by determining the likelihood of patients surviving consecutive periods (i.e., survival probability), and then multiplying each survival probability one by one to obtain the survival rate of the corresponding time period. We plotted KM curves for MRDEGs and searched for related genes with statistical differences using P < 0.05 as the threshold.

### Weighted gene co-expression network analysis (WGCNA)

WGCNA evaluates co-expression relationship between genes using the correlation coefficient of standardized expression level of each gene and defines genes with co-expression relationships as a module. Genes in the same module have similar expression levels, while those in different modules have large differences in expression levels. Through this approach, complex high-throughput data could be transformed into simple modules to some extent for dimensionality reduction. Finally, the relationship between these gene co-expression modules and clinical phenotypes could be discovered, and the biological significance of the module could be discovered. We used the WGCNA package^14^ for analysis, with a minimum module gene number of 50, a soft power setting of the optimal threshold 10, a module merge cut height setting of 0.2, and a minimum distance setting of 0.2. This method was utilized to derive co-expression modules comprising DEGs between samples in COADREAD and normal groups within the TCGA-COADREAD dataset.

### Differential gene functional enrichment analysis (FEA) and pathway enrichment analysis (PEA)

Gene ontology (GO)^15^ is an analysis approach commonly employed for conducting FEA of large-scale researches that encompass cellular component (CC), molecular function (MF), and biological process (BP) categories. In addition, Kyoto Encyclopedia of Genes and Genomes (KEGG)^16^ is a resource platform that contains information on biological pathways, genomes, illnesses, and medications. We utilized R package clusterProfiler^17^ to conduct GO annotation analysis of MRDEGs, with both P-value and FDR value (q.value) < 0.05 as the screening criteria. Benjamini–Hochberg was conducted for P-value correction to determine the statistical significance.

### Gene set enrichment analysis (GSEA)

GSEA^18^ is a way that assesses the distribution pattern of genes within a pre-defined set, by analyzing the list of genes ranked based on their association with a particular phenotype. This allows the method to determine the contribution of the gene set to the phenotype. In this study, we first assessed whether a predefined gene set exhibited significant enrichment based on the logFC value ranking of the molecules. Subsequently, clusterProfiler package was employed to conduct an enrichment analysis for all genes related to the phenotype. The GSEA was performed with the following parameters: a seed of 2020, 1000 calculations, a minimum of 10 genes per gene set, a maximum of 500 genes per gene set, and Benjamini–Hochberg correction for P-values. Molecular Signatures Database was utilized to acquire the gene set "h.all.v7.4.symbols.gmt" and conduct GSEA on expressed genes in TCGA-COADREAD dataset. Significant enrichment criteria were defined as FDR value (q.value) < 0.25 and P < 0.05.

### Macrophage diagnostic model construction

To obtain the diagnostic model for MRDEGs in TCGA-COADREAD, glmnet package^19^ was employed to execute least absolute shrinkage and selection operator (LASSO) regression on the basis of MRDEGs, with family = "binomial" as parameter, and a tenfold cross-validation was performed and run for 1000 cycles to hinder from overfitting. LASSO regression is a machine learning algorithm generally utilized in building diagnostic models today, mostly for building diagnostic models. On the basis of linear regression, by supplementing a penalty term (lambda × absolute value of slope), regularization was utilized to address the occurrence of overfitting in the curve fitting process. The model's generalization ability is also improved.

$$\mathrm{risk\,\,Score }= \sum_{{\text{i}}}\mathrm{Coefficient }\left({\mathrm{hub \,\,gene}}_{{\text{i}}}\right)\times \mathrm{mRNA \,\,Expression }\left(\mathrm{hub\, }{{\text{gene}}}_{{\text{i}}}\right).$$

Subsequently, we extracted the penalty coefficients (lambda) of the obtained MRDEGs in LASSO regression diagnostic model, followed by computing the risk scores of MRDEGs diagnostic model on the basis of MRDEGs, i.e. riskScore.

### Prognostic clinical analysis

Cox regression models were constructed for the expression of key genes and clinical variables T-stage, N-stage, and M-stage in TCGA-COADREAD dataset to evaluate the clinical prognostic value of target genes in CRC. We implemented Uni/multi-factor Cox regression analyses, built nomogram plots depending on single-factor Cox regression analysis results, and predicted CRC patient survival at 1-, 3-, and 5-year intervals. A nomogram plot is a type of plot that predicts the probability of an event based on the total score calculated from multiple independent variables. We visualized the Cox regression results, illustrated the grouping of each sample in Cox regression model in accordance with risk scores and survival outcomes, and analyzed molecular expression of prognostic MRDEGs in each group by risk factor plots.

Finally, the accuracy and discriminatory power of the column plots were evaluated utilizing calibration curves. Calibration curves are employed to evaluate how well the model predicts the actual outcome by plotting the fit of the actual probabilities and model-predicted probabilities under different scenarios. They are commonly utilized in the analysis of the fit between Cox regression model and the actual situation. We also employed decision curve analysis (DCA) to evaluate nomograms of 1-year, 3-year, and 5-year survival outcomes of CRC patients in TCGA-COADREAD dataset. R package ggDCA^20^ was employed for this analysis.

### Immune infiltration analysis (CIBERSORT)

Depending on expression matrices of human immune cell subtypes, the immune cell infiltration (ICI) status of TCGA-COADREAD dataset was assessed utilizing CIBERSORT^21^ (https://cibersort.stanford.edu/). CIBERSORT is a web version of an instrument for deconvolving expression matrices of immune cell subtypes on the basis of principle of linear support vector regression (LVR). The ICI status was assessed utilizing CIBERSORT based on the gene expression characteristics of 22 known immune cell subtypes. First, differences in infiltration of 22 immune cells in COADREAD group were analyzed, and group comparison was plotted. We then generated a heat map of correlations among immune cells and selected the immune cells with significant (P < 0.05) presence.

### COADREAD disease subtype identification

We employed Consensus Clustering^22^ to identify different subtypes of COADREAD disease in the TCGA-COADREAD dataset based on filtered MRDEGs. CC is a resampling algorithm that identifies each sample and subgroup number and verifies the clustering's rationality. ConsensusClusterPlus package in R was employed to implement CC, setting the number of clusters between 2 and 8, repeating 50 times, and drawing 80% of the total sample. We used clusterAlg = "km" and distance = "euclidean". For analyzing the differences in grouped expression of MRDEGs between samples of different disease subtypes, we performed Mann–Whitney *U* tests and considered P < 0.05 as statistically significant.

### Receiver operating characteristic (ROC) curves

In this study, ROC curves were plotted for hub genes in high and low (High/Low) score groupings in TCGA-COADREAD identified in our analysis utilizing pROC package. The ROC curve is a useful tool for evaluating the diagnostic performance of a biomarker or gene set, and area under the curve (AUC) is a common metric employed to quantify the test accuracy. Generally, an AUC value closer to 1 indicates a better diagnostic performance. ROC curves were plotted for the hub genes in both groups with high and low scores, and calculated AUC values to evaluate their diagnostic value in CRC.

### Cell cultivation and treatment

Normal human colon epithelial cell lines (FHC) and human colorectal carcinoma cell lines (HCT116) were supplied by the American Type Culture Collection. Liquid nitrogen was utilized to preserve cells. The culture medium was composed of high-glucose DMEM containing penicillin/streptomycin (100 units/ml) and fetal bovine serum (10%). Cell culture was fulfilled under the condition of 37 °C with 5% CO_2_.

### Human tissues

Eight paired CRC tissues and matched normal adjacent tissue samples were obtained from patients who underwent surgical resection at Quanzhou First Hospital Affiliated to Fujian Medical University (Fujian, China). The clinicopathological features of all samples analyzed in this study were confirmed as colorectal cancer. All specimens were frozen in liquid nitrogen. Ethical approval was confirmed by the Quanzhou First Hospital Ethics Committee, and written informed consent was obtained from each patient. All methods were performed in accordance with the relevant guidelines and regulations.

### Real-time quantitative PCR

Trizol was applied for total RNA extraction. Removal of the contaminating genomic DNA and cDNA synthesis was implemented with PrimeScript RT Reagent kit with gDNA Eraser (Takara RR047A, Japan). CFX96 Real-Time PCR detection system (Bio-Rad, Singapore) was utilized to fulfill real-time quantitative reverse transcription PCR employing TB Green Premix Ex Taq II kit. The internal reference of mRNA qPCR was glyceraldehyde-3-phosphate dehydrogenase (GAPDH). Significant differences were validated utilizing independent-sample t-test, with P < 0.05 deeming statistical significance. All primer sequences employed in the experiment are in the Table 2.

**Table 2:** Primer.

Gene name	Primer-F	Primer-R
SPP1	CTCCATTGACTCGAACGACTC	CAGGTCTGCGAAACTTCTTAGAT
MMP3	CTGGACTCCGACACTCTGGA	CAGGAAAGGTTCTGAAGTGACC
WNT5A	ATTCTTGGTGGTCGCTAGGTA	CGCCTTCTCCGATGTACTGC
TIMP1	CTTCTGCAATTCCGACCTCGT	ACGCTGGTATAAGGTGGTCTG
ADAM8	GAGGGTGAGCTACGTCCTTG	CAGCCGTATAGGTCTCTGTGT
CTSD	TGCTCAAGAACTACATGGACGC	CGAAGACGACTGTGAAGCACT
GAPDH	GTGGCAAAGTGGAGATTGTTG	AGTCTTCTGGGTGGCAGTGAT
C5AR1	TCCTTCAATTATACCACCCCTGA	ACGCAGCGTGTTAGAAGTTTTAT

### Western blot analysis

Total protein was lysed in RIPA buffer (Beyotime, Shanghai, China) in the presence of PMSF (Beyotime) and PhosSTOP (Roche, Basel, Switzerland). Western blots were carried out according to standard procedures. Antibody against MMP3 was obtained from Bio-Techne. Antibody ADAM8、TIMP1、C5AR1 was obtained from proteintech.

### Statistical analyses

R software (Version 4.1.2) was employed to process and analyze data. Independent student *t*-test and Mann–Whitney *U*-test were implemented for variables with normal distributions and non-normal distributions, respectively. Categorical variables were compared utilizing χ^2^ test or Fisher's exact test. Unless otherwise specified, spearman correlation analysis was implemented to compute correlation coefficients among different molecules, with P < 0.05 deeming statistical significance.

## Results

### Sketch of study design and GEO dataset merging

**Figure 1:**
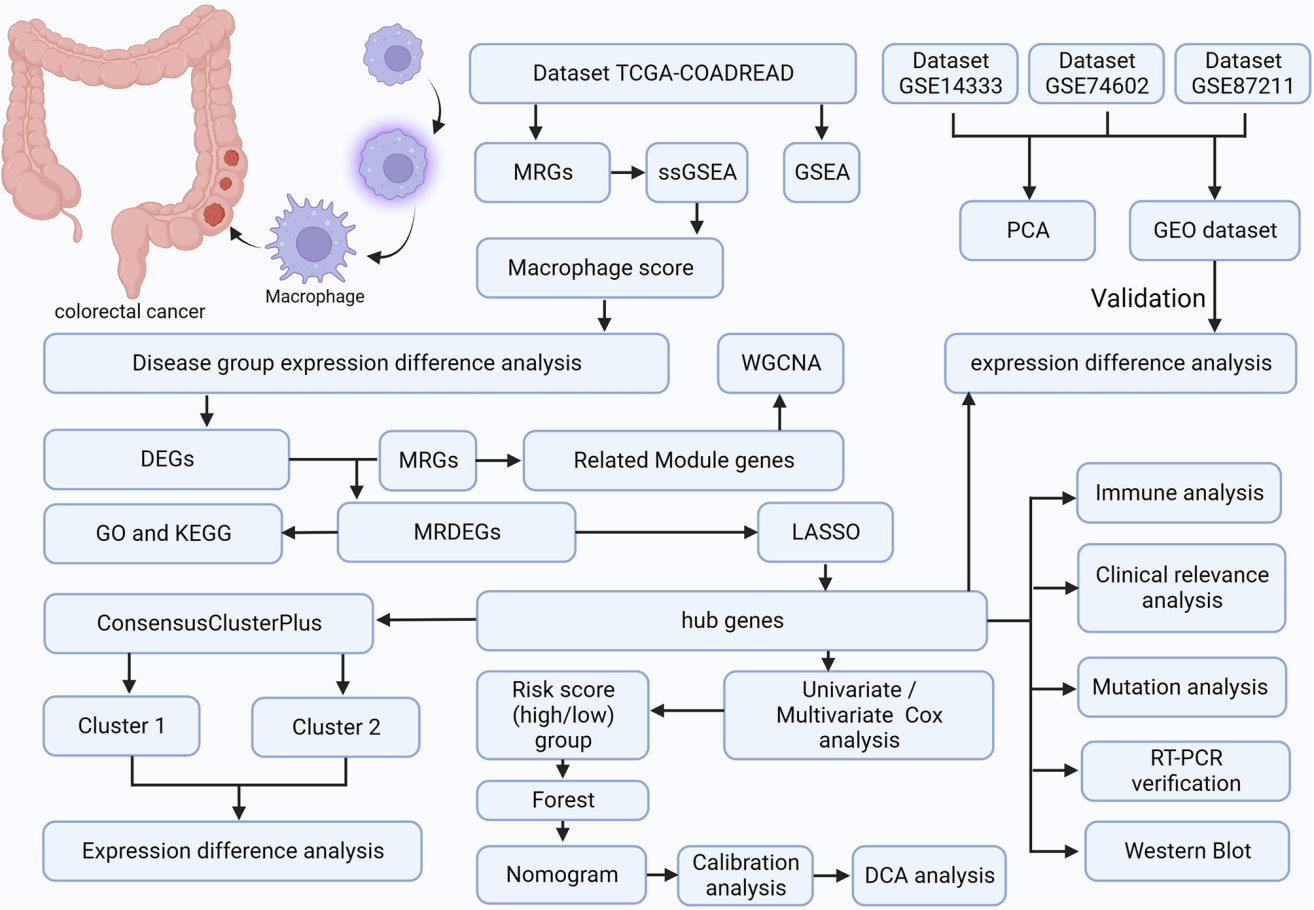
Flow diagram of overall analysis of bioinformatics approach in exploring the biological features of CRC. *DCA* decision curve analysis, *DEGs* differentially expressed genes, *GO* gene ontology, *GSEA* gene set enrichment analysis, KEGG Kyoto encyclopedia of genes and genomes, *LASSO* least absolute shrinkage and selection operator, *MRDEGs* macrophage-related DEGs, *PCA* principal component analysis, *ssGSEA* single-sample GSEA, *WGCNA* weighted gene co-expression network analysis. Created with BioRender.com.

As can be seen in the research protocol (Fig. 1), we obtained MS by phenotypic scoring of MRGs on the dataset TCGA-COADREAD, and then categorized the samples into groups with high/low scores in accordance with median phenotypic scores. Then differential expression analysis was implemented in two groups to obtain DEGs, which were intersected with MRGs to obtain MRDEGs, and MRDEGs were intersected with weighted gene co-expression module related genes to obtain key genes by LASSO model screening, and consistency clustering analysis, cox analysis, immuno-infiltration analysis, mutation analysis, clinical relevance analysis, etc. were carried out. Finally, the datasets GSE14333, GSE74602, GSE87211 were used for external validation of expression analysis, and real-time quantitative PCR were used for validation of bioinformatics results.

Then, we removed the batch effect for CRC datasets GSE14333, GSE74602 and GSE87211 to obtain the merged dataset GEO dataset, and compared the datasets before and after batch effect removal by distribution box line plots and PCA plots (Fig. S1). The results of the distribution box line plots and PCA plots showed that the batch effect of the samples in GEO dataset is largely eliminated after the batch removal process.

### Analysis of DEGs associated with CRC

**Figure 2:**
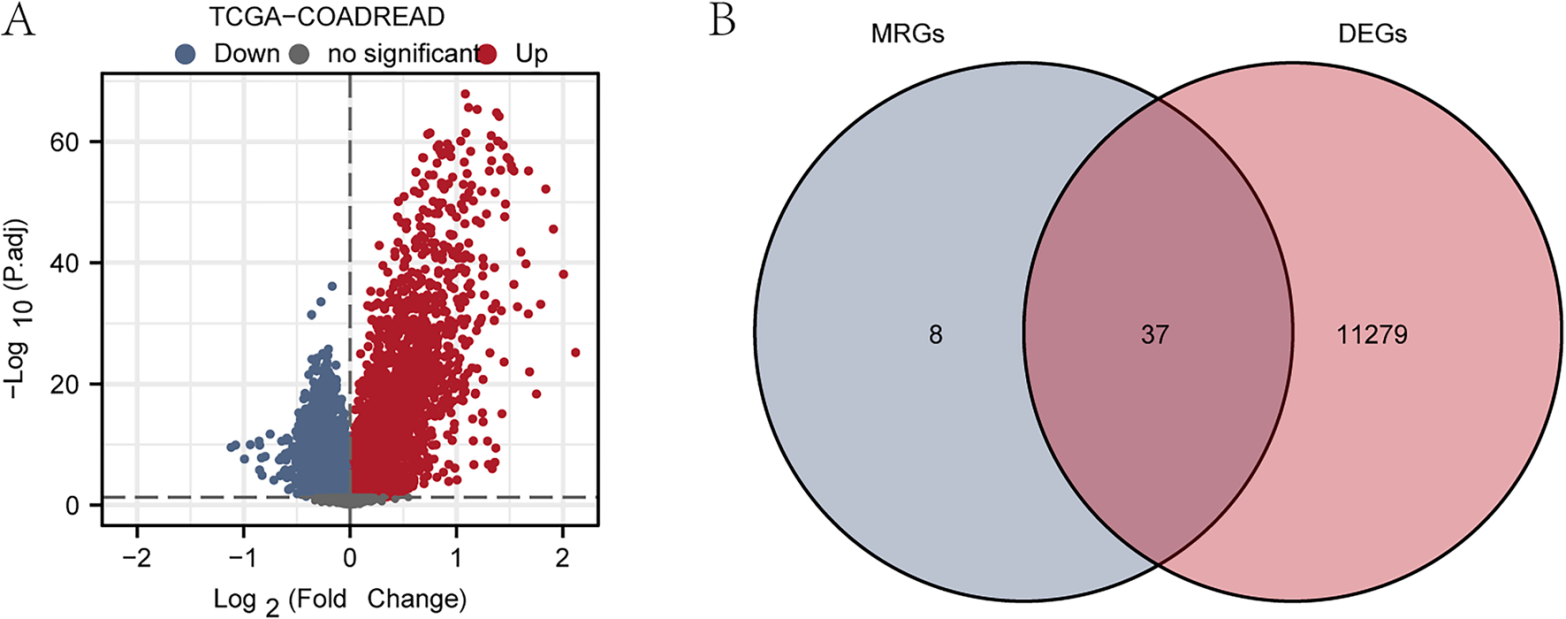
Analysis of differential genes in CRCTCGA-COADREAD dataset. (**A**) Volcano plot of differential genes. (**B**) Venn diagram of prognostic molecular DEGs and MRGs. *DEGs* differentially expressed genes, *MRGs* macrophage-related genes.

To analyze the DEGs between groups with high/low MS scores of COADREAD patients in TCGA-COADREAD dataset, differential analysis was fulfilled on TCGA-COADREAD dataset utilizing limma package to obtain DEGs of the data. The results are as follows: with |logFC|> 0 and P < 0.05 as the thresholds, there were 11,316 genes identified in TCGA-COADREAD dataset, including 4074 up-regulated genes with logFC > 0 and 7242 down-regulated genes with logFC < 0. According to differential analysis results of this dataset, a volcano plot was plotted (Fig. 2A).

To identify Macrophage-related differentially expressed genes (MRDEGs), we initially conducted a univariate Cox regression analysis on a set of 637 MRGs (Macrophage-related genes). Among these genes, we selected those with a p-value < 0.05, resulting in a final set of 45 MRGs that exhibit prognostic significance. Detailed information about these MRGs can be found in Table S2. Subsequently, we compared these 45 MRGs with all the differentially expressed genes (DEGs) derived from the TCGA-COADREAD dataset, specifically focusing on genes with |logFC|> 0 and a P-value < 0.05. The overlapping genes from this analysis yielded a total of 37 MRDEGs. To visually represent the intersection results, we created a Venn diagram (Fig. 2B).

The expression differences between various sample groups in TCGA-COADREAD dataset were analyzed, and R package pheatmap was employed to plot heat maps to show the analysis results. We selected the differential analysis results of 37 MRDEGs for heat map display, with these results displaying in Table S1.

### FEA (GO) and PEA (KEGG) of MRDEGs

**Figure 3:**
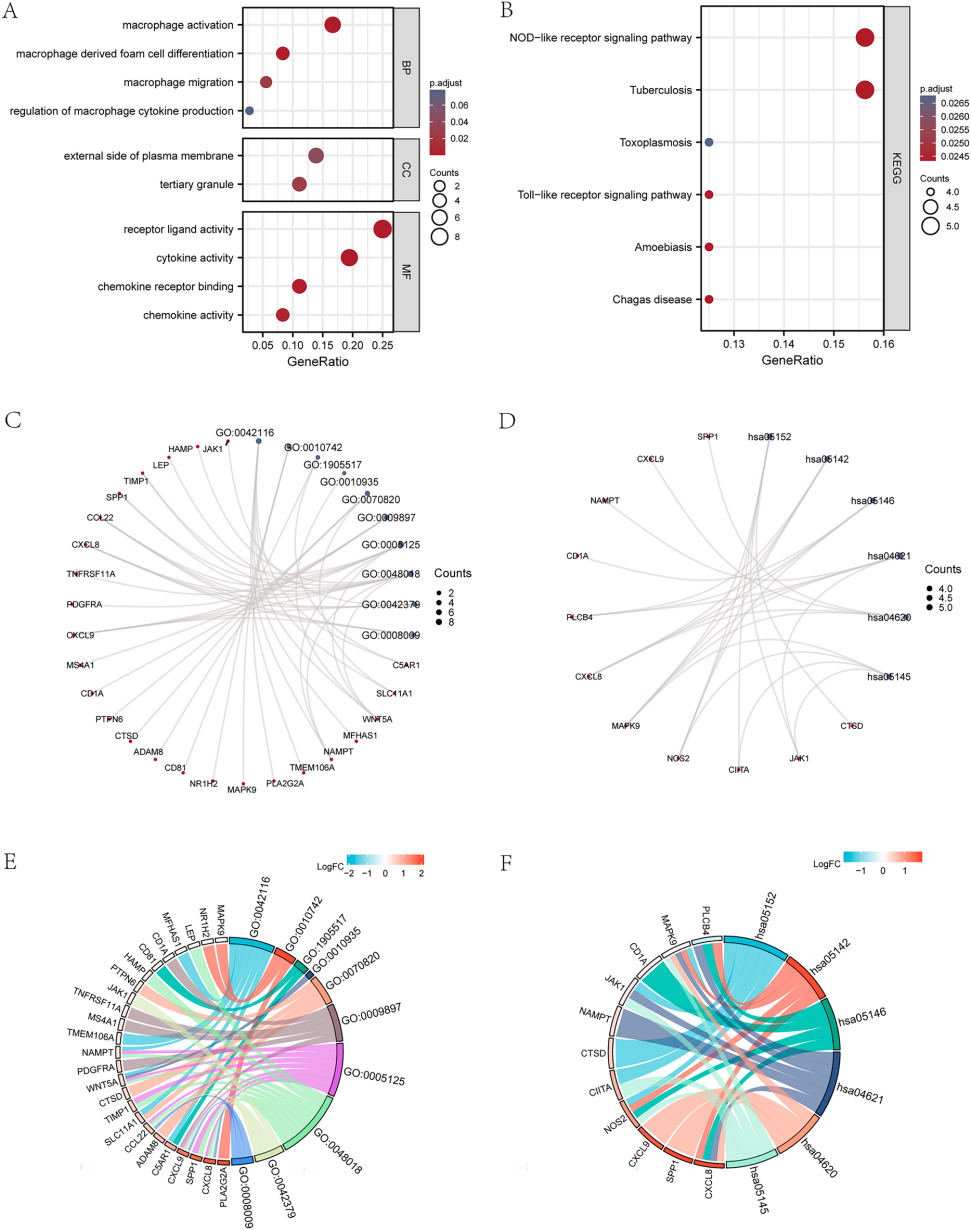
GO and KEGG analyses of MRDEGs. (**A**,**B**) Bubble chart of GO (**A**) and KEGG (**B**) analyses of MRDEGs. (**C**,**D**) Circular network diagram of GO (**C**) and KEGG (**D**) analyses of MRDEGs. (**E**,**F**) Chord diagram of GO (**E**) and KEGG (**F**) analyses of MRDEGs. Both P.value and FDR value (q.value) less than 0.05 were taken as the screening criteria of GO and KEGG enrichment entries.

For the purpose of analyzing BP, CC, MF, biological pathways, and their association with colon cancer of 37 MRDEGs, we first performed GO (Table 3) and KEGG (Table 4) enrichment analyses on MRDEGs. P.value < 0.05 served as screening criteria of enrichment entries, and FDR value (q.value) < 0.05 was deemed to statistically significant. We showed the results of GO FEA and KEGG PEA in bubble charts (Fig. 3A,B), circular network diagrams (Fig. 3C,D), and chord diagrams (Fig. 3E,F).

**Table 3:** GO enrichment analysis results of MRDEGs.

Ontology	ID	Description	GeneRatio	BgRatio	pvalue	p.adjust	qvalue
BP	GO:0050727	Regulation of inflammatory response	12/36	485/18,670	5.86e-11	1.22e-07	6.52e-08
BP	GO:0006869	Lipid transport	10/36	365/18,670	1.17e-09	1.22e-06	6.48e-07
BP	GO:0010876	Lipid localization	10/36	400/18,670	2.82e-09	1.96e-06	1.04e-06
BP	GO:0070372	Regulation of ERK1 and ERK2 cascade	9/36	300/18,670	4.08e-09	2.13e-06	1.13e-06
BP	GO:0070371	ERK1 and ERK2 cascade	9/36	317/18,670	6.59e-09	2.75e-06	1.46e-06
CC	GO:0070820	Tertiary granule	4/36	164/19,717	2.21e-04	0.028	0.020
CC	GO:0009897	External side of plasma membrane	5/36	393/19,717	6.95e-04	0.044	0.032
CC	GO:0034774	Secretory granule lumen	4/36	321/19,717	0.003	0.069	0.051
CC	GO:0042581	Specific granule	3/36	160/19,717	0.003	0.069	0.051
CC	GO:0060205	Cytoplasmic vesicle lumen	4/36	338/19,717	0.003	0.069	0.051
MF	GO:0005125	Cytokine activity	7/36	220/17,697	2.56e-07	3.16e-05	2.13e-05
MF	GO:0048018	Receptor ligand activity	9/36	482/17,697	3.75e-07	3.16e-05	2.13e-05
MF	GO:0042379	Chemokine receptor binding	4/36	66/17,697	9.50e-06	5.35e-04	3.60e-04
MF	GO:0008009	Chemokine activity	3/36	49/17,697	1.34e-04	0.006	0.004
MF	GO:0045236	CXCR chemokine receptor binding	2/36	11/17,697	2.19e-04	0.007	0.005

*MRDEGs* macrophage-related differentially expressed genes, *GO* gene ontology, *BP* biological process, *CC* cellular component, *MF* molecular function.

**Table 4:** KEGG enrichment analysis results of MRDEGs.

Ontology	ID	Description	GeneRatio	BgRatio	pvalue	p.adjust	qvalue
KEGG	hsa05152	Tuberculosis	5/32	180/8076	6.43e-04	0.024	0.019
KEGG	hsa05142	Chagas disease	4/32	102/8076	6.57e-04	0.024	0.019
KEGG	hsa05146	Amoebiasis	4/32	102/8076	6.57e-04	0.024	0.019
KEGG	hsa04621	NOD-like receptor signaling pathway	5/32	181/8076	6.59e-04	0.024	0.019
KEGG	hsa04620	Toll-like receptor signaling pathway	4/32	104/8076	7.07e-04	0.024	0.019

*MRDEGs* macrophage-related differentially expressed genes, *KEGG* Kyoto encyclopedia of genes and genomes.

### GSEA of CRC dataset

**Figure 4:**
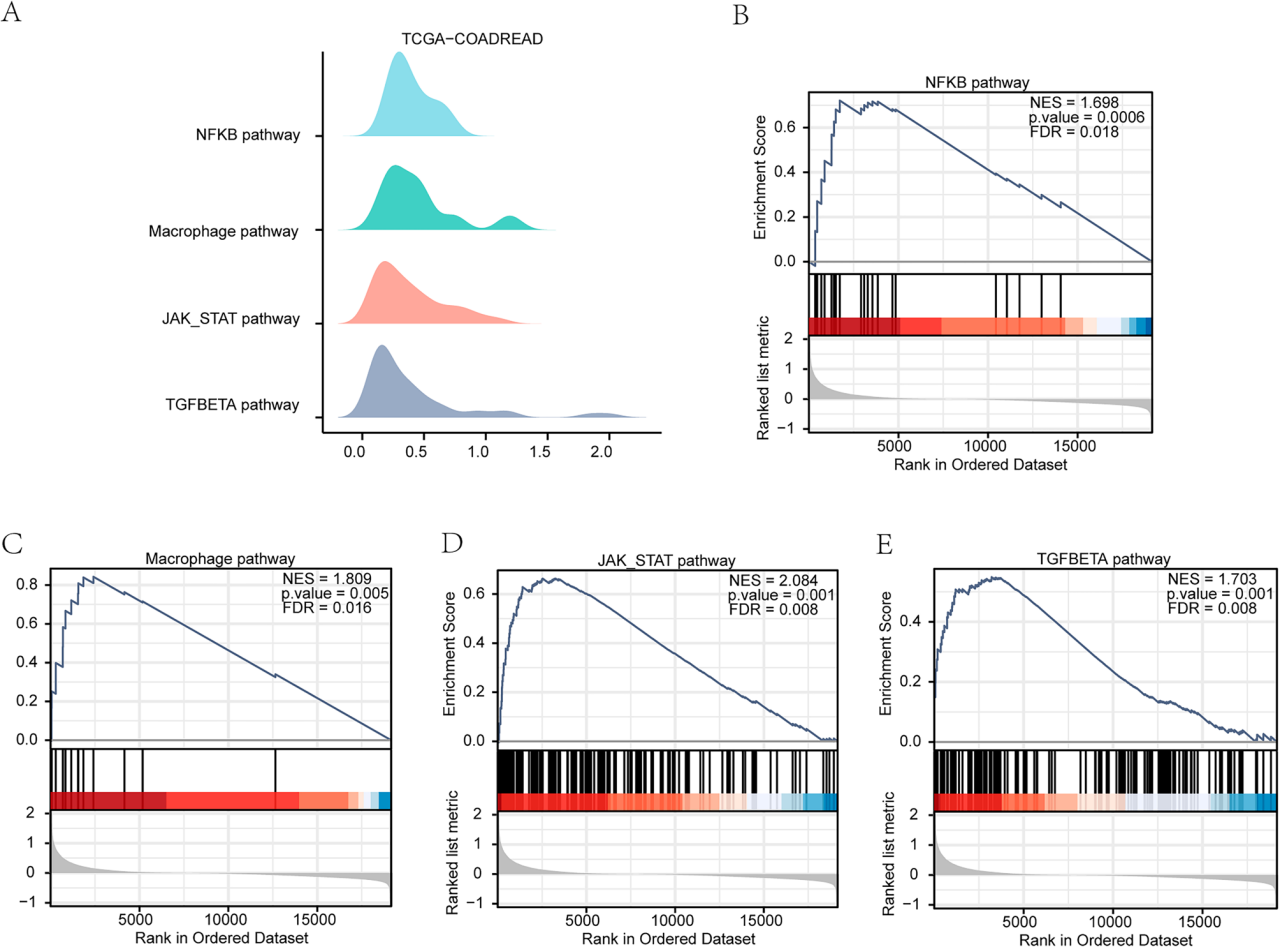
GSEA of TCGA-COADREAD dataset. (**A**) Four main biological features of GSEA in the TCGA-COADREAD dataset. (**B-E**) Differential genes in TCGA-COADREAD dataset showed significant enrichment in NFKB pathway, Macrophage pathway, JAK_STAT pathway, TGFBETA pathway. Blue represents group with low scores group and red represents group with high scores. *GSEA* gene set enrichment analysis. FDR value (q.value) less than 0.25 and P-value less than 0.05 served as screening criteria of significant enrichment for GSEA.

We studied how gene expression levels relate to colon cancer by looking at the differences in gene expression, BP, CC, and MF between two groups of CRC patients (high/low scores) in TCGA-COADREAD dataset using GSEA. All genes in TCGA-COADREAD showed significant enrichment in pathways (Fig. 4) like NFKB pathway, Macrophage pathway, JAK_STAT pathway, TGFBETA pathway, etc. (Table 5).

**Table 5:** GSEA analysis of TCGA-COADREAD.

Description	setSize	enrichmentScore	NES	pvalue	p.adjust	qvalues
REACTOME_SIGNALING_BY_INTERLEUKINS	456	0.701298128	2.393264504	0.001027749	0.012502634	0.008280184
REACTOME_GPCR_LIGAND_BINDING	460	0.629681172	2.14886594	0.001029866	0.012502634	0.008280184
REACTOME_NEUTROPHIL_DEGRANULATION	476	0.682547961	2.330513729	0.001029866	0.012502634	0.008280184
WP_VEGFAVEGFR2_SIGNALING_PATHWAY	429	0.521171688	1.770735811	0.001035197	0.012502634	0.008280184
REACTOME_G_ALPHA_I_SIGNALLING_EVENTS	401	0.605939472	2.050456534	0.001036269	0.012502634	0.008280184
REACTOME_CLASS_A_1_RHODOPSIN_LIKE_RECEPTORS_	328	0.697306527	2.329167117	0.001057082	0.012502634	0.008280184
WP_PI3KAKT_SIGNALING_PATHWAY	339	0.583911284	1.952058028	0.001057082	0.012502634	0.008280184
NABA_SECRETED_FACTORS	342	0.668712195	2.232364181	0.001060445	0.012502634	0.008280184
KEGG_PATHWAYS_IN_CANCER	325	0.522291083	1.743352174	0.001062699	0.012502634	0.008280184
WP_NUCLEAR_RECEPTORS_METAPATHWAY	317	0.431864318	1.439679141	0.001066098	0.012502634	0.008280184
REACTOME_METABOLISM_OF_CARBOHYDRATES	293	0.459825426	1.527002163	0.001072961	0.012502634	0.008280184
WP_FOCAL_ADHESIONPI3KAKTMTORSIGNALING_PATHWAY	303	0.63321308	2.103597705	0.001072961	0.012502634	0.008280184
REACTOME_EXTRACELLULAR_MATRIX_ORGANIZATION	301	0.777275698	2.578891043	0.001074114	0.012502634	0.008280184
NABA_CORE_MATRISOME	274	0.766890111	2.535010872	0.001082251	0.012502634	0.008280184
WP_IL18_SIGNALING_PATHWAY	272	0.687058296	2.268978836	0.001082251	0.012502634	0.008280184

*GSEA* gene set enrichment analysis.

### WGCNA to screen co-expression modules in the dataset TCGA-COADREAD

**Figure 5:**
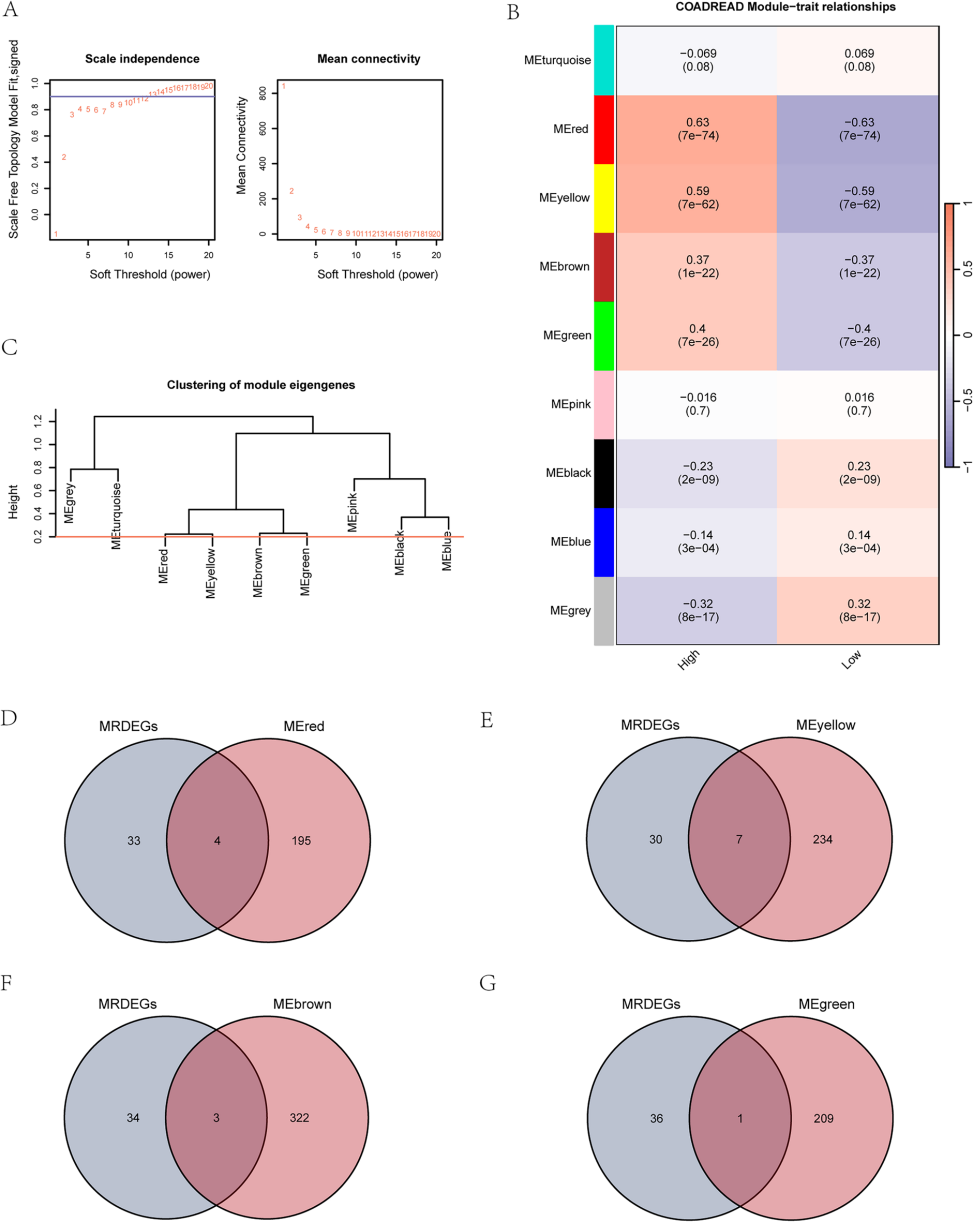
WGCNA to identify co-expression modules in TCGA-COADREAD dataset. (**A**) The unscaled network display of sample modules. (**B**) The correlation analysis results of DEGs clustering modules. (**C**) The module aggregation results of DEGs. (**D-G**) Venn diagrams of DEGs in four modules MEred, MEyellow, MEbrown, and MEgreen. *MRDEGs* macrophage-related DEGs, *WGCNA* weighted gene co-expression network analysis.

We performed WGCNA on the DEGs in colon cancer patients with high/low scores in TCGA-COADREAD dataset to screen for co-expression modules. In the WGCNA process, we first clustered colon cancer patients with high/low scores in TCGA-COADREAD dataset using a clustering tree and labeled grouping information (without setting cut height). We set a screening criterion of 50 to identify the best number of modules. The DEGs of CRC patients with high/low scores in TCGA-COADREAD dataset were aggregated into nine modules (MEturquoise, MEred, MEyellow, MEbrown, MEgreen, MEpink, MEdarkgrey, MEblack, MEblueMEgrey) (Fig. 5A). The DEGs in colon cancer patients with high/low scores in TCGA-COADREAD dataset were clustered again and the relationship between genes and corresponding new modules was visualized. Finally, depending on expression patterns of module genes and grouping information of two groups in TCGA-COADREAD dataset, we obtained nine modules (MEturquoise, MEred, MEyellow, MEbrown, MEgreen, MEpink, MEdarkgrey, MEblack, MEblueMEgrey) and their correlation with CRC patients with high/low scores in TCGA-COADREAD dataset (Fig. 5B). Then we merged modules with a cut height set to 0.2 and clipped and merged modules with a cut height below 0.2 (Fig. 5C).

Firstly, we analyzed four modules (MEred, MEyellow, MEbrown, MEgreen) containing DEGs that show significant statistical differences. (P < 0.05, correlation absolute value ≥ 0.3) and correlations with CRC patients with high/low scores in TCGA-COADREAD dataset among nine modules (excluding useless gray module: MEgrey). Firstly, we took intersections between MRDEGs in colon cancer patients with high/low scores in TCGA-COADREAD dataset with DEGs contained in four modules respectively and drew Venn diagrams (Figs. 5D–G) to obtain module MRDEGs. As shown in Fig. 5, we obtained a total of 15 MRDEGs (SLC11A1, SPP1, CXCL9, MMP3, CXCL8, CIITA, C5AR1, WNT5A, PDGFRA, FABP4, TIMP1, CCL22, CTSD, ADAM8, MS4A1).

**Figure 6:**
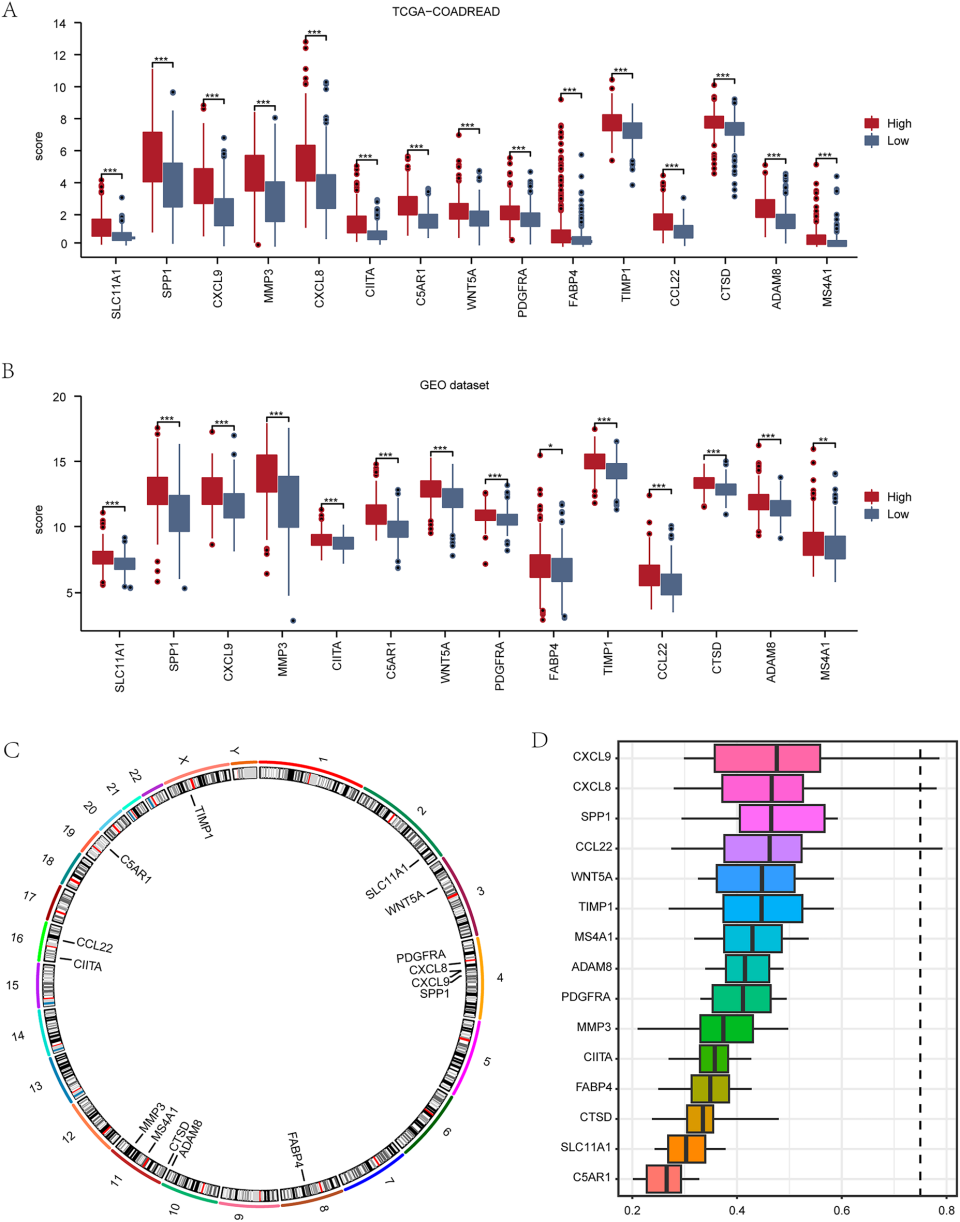
Expression of MRDEGs in CRC dataset. (**A**,**B**) The grouping comparison chart of MRDEGs in CRC patients with high/low scores in TCGA-COADREAD (**A**) and GEO (**B**) datasets is shown in the MRDEGs diagnostic model. (**C**) Chromosome location map of MRDEGs. (**D**) Chromosome location map of MRDEGs. Blue represents high score group, and red represents low score group. *P < 0.05 refers to significant difference, **P < 0.01 to high significant difference, ***P < 0.001 to extremely significant difference. *MRDEGs* macrophage-related differentially expressed genes.

In this study, the expression levels of 15 MRDEGs (SLC11A1, SPP1, CXCL9, MMP3, CXCL8, CIITA, C5AR1, WNT5A, PDGFRA, FABP4, TIMP1, CCL22, CTSD, ADAM8, MS4A1) were analyzed in two groups of colon cancer patients with high/low scores in both TCGA-COADREAD (Fig. 6A) and GEO datasets (Fig. 6B). The results showed that the expression levels of all 15 MRDEGs were statistically significantly different (P < 0.001) in TCGA-COADREAD dataset, whereas in GEO dataset, 12 MRDEGs (SLC11A1, SPP1, CXCL9, MMP3, CXCL8, CIITA, C5AR1, WNT5A, PDGFRA, TIMP1, CCL22, CTSD, ADAM8) exhibited significant differences (P < 0.001) between the two groups.

We then annotated the positions of 15 MRDEGs on human chromosomes and visualized them using circle diagrams (Fig. 6C). As shown in the figure: gene WNT5A is located on chromosome 3 and SLC11A1 is located on chromosome 2. We then performed friends analysis on 15 MRDEGs and visualized them using a plot (Fig. 6D). Then we generated ROC curves for 15 MRDEGs (SLC11A1, SPP1, CXCL9, MMP3, CXCL8, CIITA, C5AR1, WNT5A, PDGFRA, FABP4, TIMP1, CCL22, CTSD, ADAM8, MS4A1) in both TCGA-COADREAD and GEO datasets, demonstrating the association between high/low scores of these genes and CRC patients. (Figs. S2 and S3).

### Correlation analysis between hub genes and MS

To explore the relationship between 15 MRDEGs (SLC11A1, SPP1, CXCL9, MMP3, CXCL8, CIITA, C5AR1, WNT5A, PDGFRA, FABP4, TIMP1, CCL22, CTSD, ADAM8, MS4A1) and the macrophage score, we created a scatter plot (Fig. S4) to visualize their correlation. The results indicated that a subset of MRDEGs (C5AR1, CXCL8, CIITA, CXCL9, ADAM8, CCL22, SLC11A1, MMP3) exhibited a moderate level of correlation with the macrophage score (0.5 < r < 0.8). Conversely, the remaining MRDEGs (SPP1, CTSD, TIMP1, MS4A1, PDGFRA, WNT5A, FABP4) displayed a weak correlation with the macrophage score (0.3 < r < 0.5).

### Construction of the diagnostic model for MRDEGs

**Figure 7:**
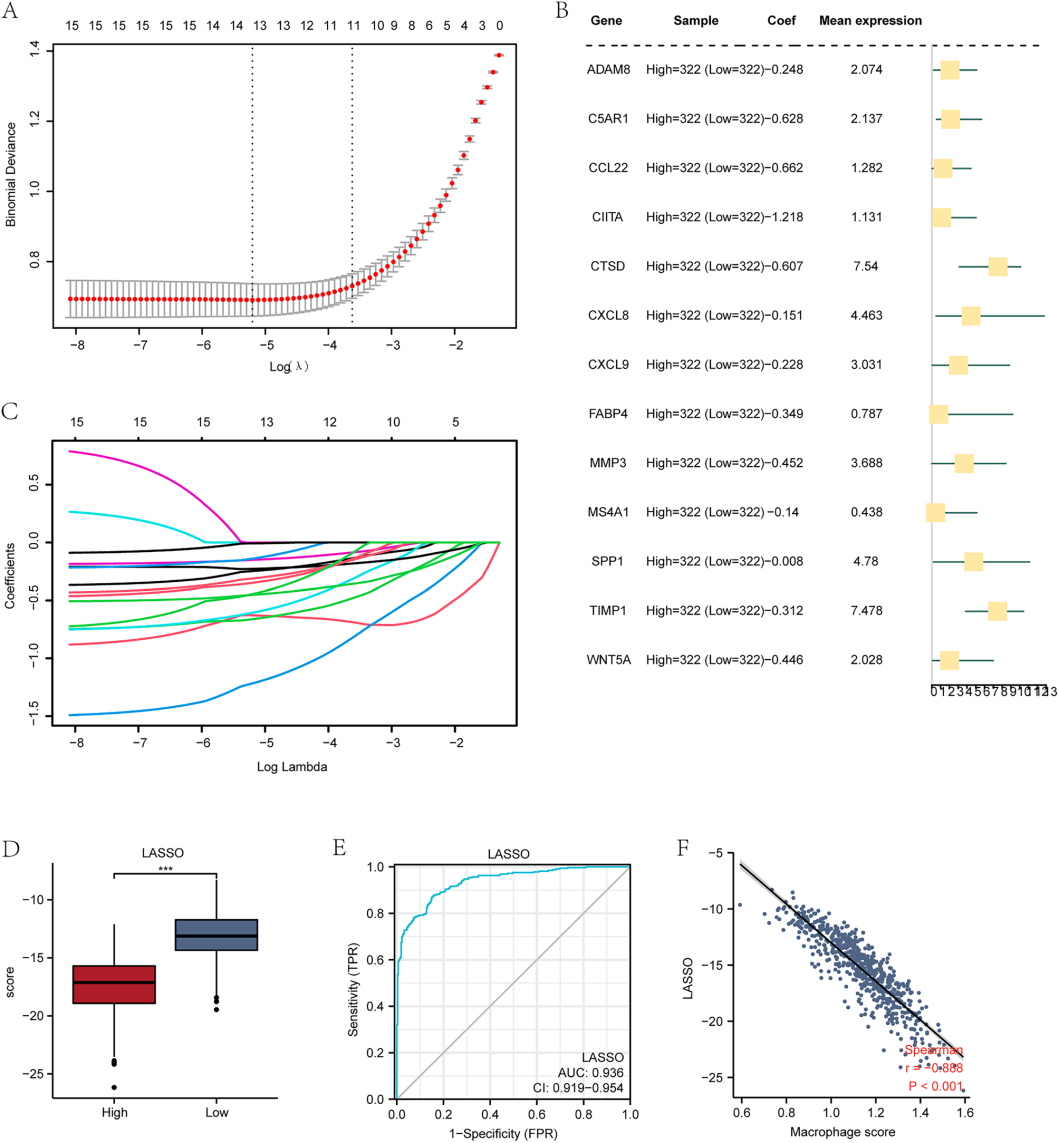
Establishment of the MRDEGs diagnostic model. (A) LASSO regression diagnostic model diagram of MRDEGs. (**B**) Forest plot results of MRDEGs in diagnostic model. (**C**) LASSO variable trajectory chart of MRDEGs diagnostic model. (**D**) Group comparison chart of MRDEGs diagnostic model. (**E**) ROC analysis of MRDEGs diagnostic model. (**F**) Scatter plot of correlation of MRDEGs diagnostic model with macrophage score. *LASSO* least absolute shrinkage and selection operator, *MRDEGs* macrophage-related differentially expressed genes.

To determine the diagnostic value of 15 MRDEGs in TCGA-COADREAD dataset, a MRDEGs diagnostic model was constructed utilizing LASSO regression analysis (Fig. 7A). Then we visualized the expression of MRDEGs in different groups through a forest plot (Fig. 7B). According to Fig. 6B, there are a total of 13 MRDEGs (ADAM8, C5AR1, CCL22, CIITA, CTSD, CXCL8, CXCL9, FABP4, MMP3, MS4A1, SPP1, TIMP1, WNT5A) in the MRDEGs diagnostic model we constructed. LASSO regression is a type of linear regression that includes a penalty term to mitigate overfitting and enhance the model's ability to generalize. We visualized the LASSO variable trajectory based on LASSO regression results (Fig. 7C), which showed that gene expression changes with lambda coefficient (log) of LASSO penalty term. As lambda decreases, the number of genes with a coefficient of zero gradually increases. Differential expression analysis of MRDEGs diagnostic model of CRC patients with high/low scores in TCGA-COADREAD dataset was conducted (Fig. 7D), and the two groups exhibited marked differences in expression levels of MRDEGs diagnostic model (P < 0.001).

A ROC curve was drawn for MRDEGs diagnostic model of CRC patients with high/low scores in TCGA-COADREAD dataset. As shown in Fig. 7E, MRDEGs diagnostic model (AUC = 0.936) has high diagnostic value for colon cancer patients in TCGA-COADREAD dataset. The correlation between MRDEGs diagnostic model and MS was illustrated by creating a scatter plot (Fig. 7F). The plot indicates a statistically significant difference between LASSO and MS (P < 0.001).

### Prognostic performance of MRDEGs

To probe the correlation of expression of 13 MRDEGs (ADAM8, C5AR1, CCL22, CIITA, CTSD, CXCL8, CXCL9, FABP4, MMP3, MS4A1, SPP1, TIMP1, WNT5A) with the incidence of CRC, univariate/multivariate Cox regression analysis was implemented on expression levels of MRDEGs and clinical variables M stage, N stage, and T stage with prognostic clinical relationship in TCGA-COADREAD dataset. The analysis result illustrated a correlation between expression levels of MRDEGs and clinical variables M stage, N stage, and T stage with prognostic clinical relationship. In this study, the clinical data of COADREAD patients acquired from TCGA-COADREAD dataset was also statistically analyzed (Table 6).

**Table 6:** Univariate and multivariate cox regression.

Characteristics	Total (N)	Univariate analysis		Multivariate analysis	
		Hazard ratio (95% CI)	P value	Hazard ratio (95% CI)	P value
T stage	640				
T1&T2	131	Reference			
T3	435	2.047 (1.090–3.842)	**0.026**	1.500 (0.666–3.379)	0.328
T4	74	6.148 (3.045–12.415)	** < 0.001**	2.577 (1.013–6.554)	**0.047**
N stage	639				
N0	367	Reference			
N1	153	1.774 (1.131–2.781)	**0.013**	1.318 (0.776–2.237)	0.307
N2	119	3.873 (2.588–5.796)	** < 0.001**	2.487 (1.490–4.151)	** < 0.001**
M stage	563				
M0	474	Reference			
M1	89	3.989 (2.684–5.929)	** < 0.001**	2.135 (1.303–3.500)	**0.003**
ADAM8	643				
High	321	Reference			
Low	322	0.601 (0.423–0.853)	**0.004**	0.639 (0.391–1.042)	0.073
C5AR1	643				
High	321	Reference			
Low	322	0.715 (0.504–1.012)	0.058	0.604 (0.351–1.039)	0.069
CCL22	643				
High	322	Reference			
Low	321	1.871 (1.299–2.694)	** < 0.001**	1.531 (0.934–2.512)	0.091
CIITA	643				
High	322	Reference			
Low	321	1.628 (1.137–2.332)	**0.008**	1.130 (0.662–1.931)	0.654
CTSD	643				
High	322	Reference			
Low	321	0.769 (0.543–1.088)	0.137		
CXCL8	643				
High	321	Reference			
Low	322	1.525 (1.068–2.176)	**0.020**	1.080 (0.658–1.775)	0.760
CXCL9	643				
High	322	Reference			
Low	321	1.595 (1.119–2.275)	**0.010**	1.238 (0.752–2.038)	0.402
FABP4	643				
High	321	Reference			
Low	322	0.583 (0.408–0.832)	**0.003**	0.824 (0.523–1.298)	0.403
MMP3	643				
High	321	Reference			
Low	322	1.623 (1.133–2.325)	**0.008**	1.404 (0.859–2.293)	0.176
MS4A1	643				
High	321	Reference			
Low	322	1.729 (1.204–2.484)	**0.003**	1.952 (1.208–3.152)	**0.006**
SPP1	643				
High	322	Reference			
Low	321	0.774 (0.547–1.096)	0.149		
TIMP1	643				
High	322	Reference			
Low	321	0.645 (0.454–0.917)	**0.015**	0.830 (0.541–1.275)	0.395
WNT5A	643				
High	321	Reference			
Low	322	1.496 (1.046–2.138)	**0.027**	1.417 (0.892–2.253)	0.140

*CI* confidence interval.

Significant values are in bold.

**Figure 8:**
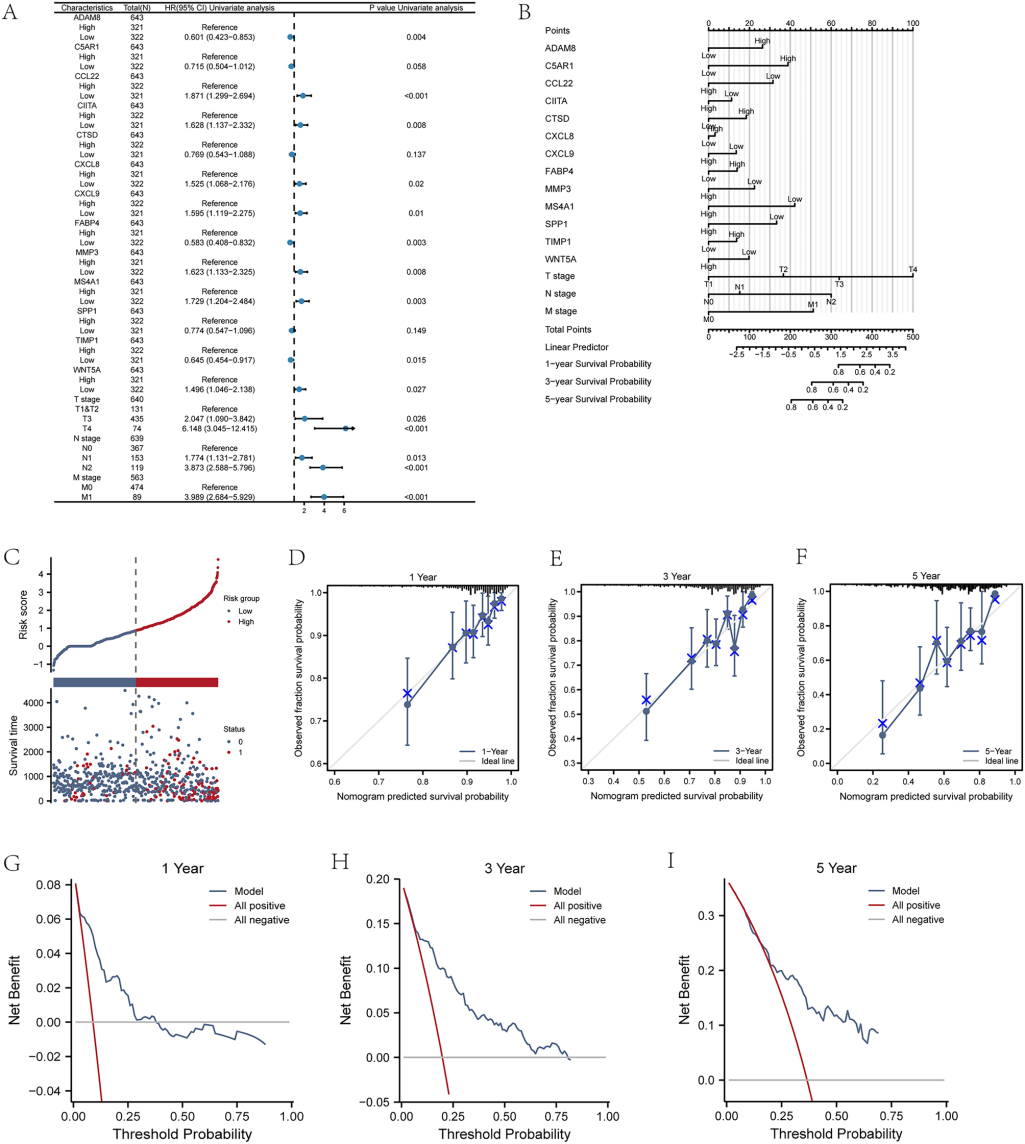
Prognostic performance of MRDEGs. (**A**-**C**) Forest plot (**A**), nomogram chart (**B**), and risk factor chart (**C**) of univariate regression analysis of MRDEGs. (**D-F**) Calibration curve of Cox regression prognostic model for 1-year (**D**), 3-year (**E**), and 5-year (**F**). (**G-I**) Decision curve analysis chart of Cox regression prognostic model for 1-year (**G**), 3-year (**H**), and 5-year (**I**).

A forest plot (Fig. 8A) was utilized to present univariate/multivariate Cox regression analysis results (Table 6). Subsequently, the prognostic ability of Cox regression model was assessed through nomogram analysis, and a nomogram chart was generated (Fig. 8B). Additionally, in Cox regression model, a risk factor chart was employed to visualize grouping of risk factors (Fig. 8C).

In our research, calibration analysis was implemented on the variables in univariate/multivariate Cox regression models for 1-, 3-, and 5-year periods, and results were presented in calibration curve charts (Fig. 8D–F). Furthermore, DCA was implemented to appraise the clinical utility of Cox regression prognostic model constructed for 1-, 3-, and 5-year periods and presented the results (Fig. 8G–I).

**Figure 9:**
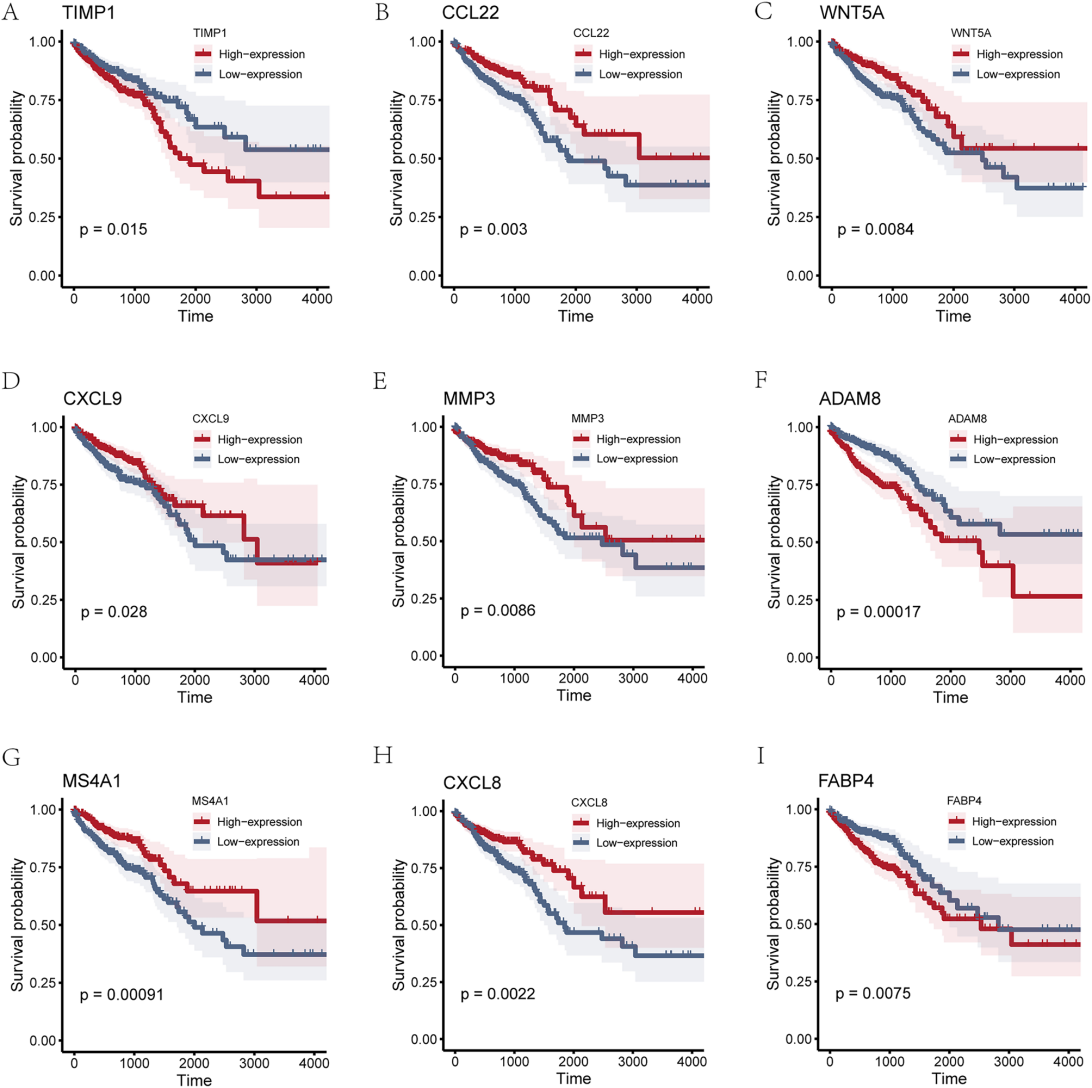
Prognostic performance of MRDEGs KM curve (OS) analysis. (**A**-**I**) The KM curve (OS) of MRDEGs was used to perform prognostic analysis. The low-expression group of colon cancer patient samples is represented by a blue line, while the high-expression group of colon cancer patient samples is represented by a red line. OS refers to overall survival, and the KM curve refers to the Kaplan–Meier curve. P < 0.05 refers to significant difference, P < 0.01 to high significant difference, and P < 0.001 to extremely high significant difference.

We drew prognostic survival KM curves for 13 MRDEGs (ADAM8, C5AR1, CCL22, CIITA, CTSD, CXCL8, CXCL9, FABP4, MMP3, MS4A1, SPP1, TIMP1, WNT5A) in TCGA-COADREAD dataset. It showed that only 9 MRDEGs (Fig. 9) met the requirements when each of the 13 MRDEGs was drawn one by one with a prognostic survival KM curve using P < 0.05 as the standard for statistically significant correlation molecules.

### Construction of COADREAD-related disease subtypes

**Figure 10:**
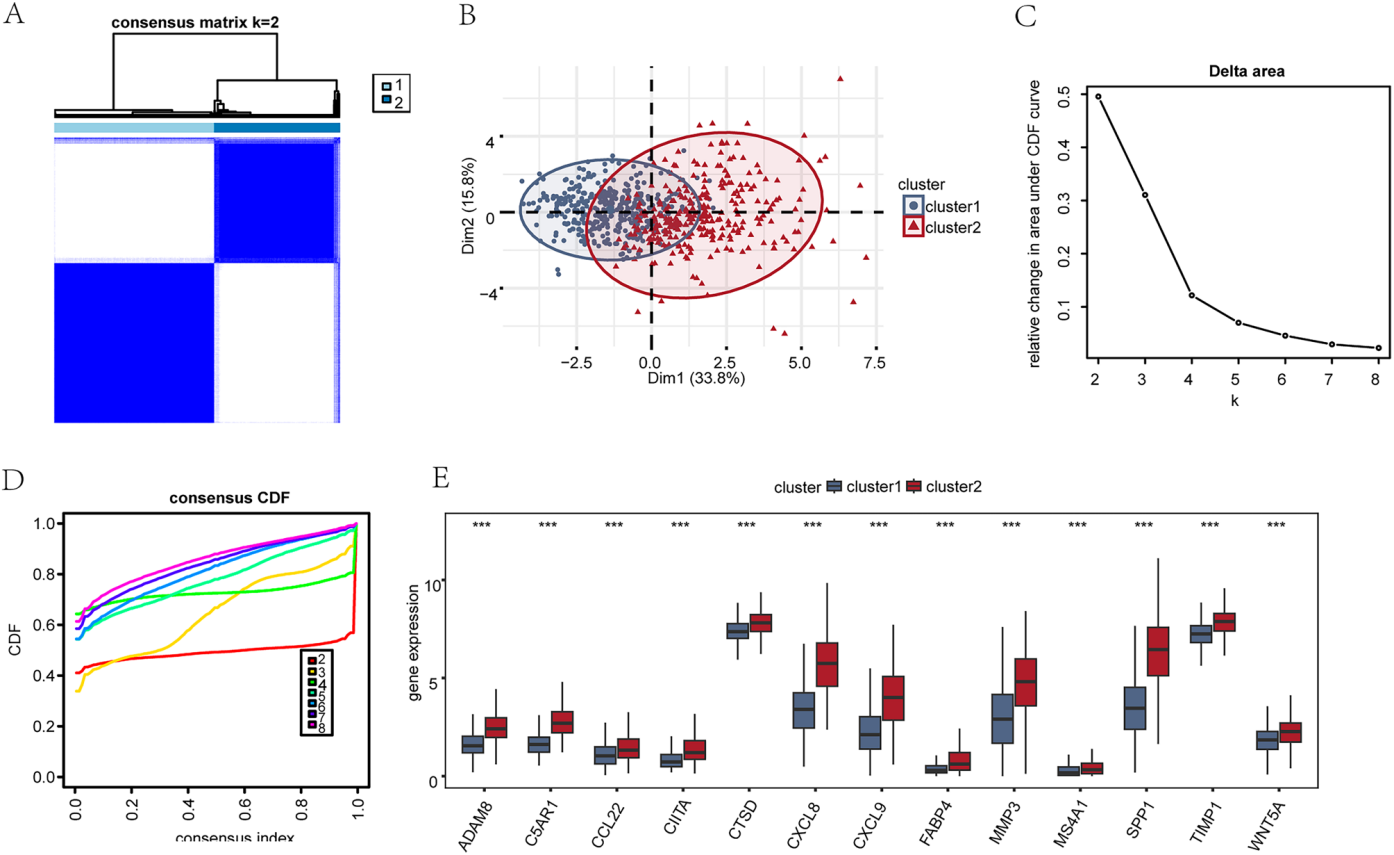
Construction of correlated disease subtypes of COADREAD. (**A**) Consistency clustering (K = 2) result of COADREAD disease in TCGA-COADREAD dataset. (**B**) PCA results of two COADREAD disease subtypes (cluster1 and cluster2) in TCGA-COADREAD dataset. (**C**,**D**) Delta plot (**C**) and cumulative distribution function (CDF) plot (**D**) of different numbers of clusters in consistency clustering. (**E**) Group comparison of MRDEGs in different subtypes of COADREAD disease in TCGA-COADREAD dataset. ***represents P < 0.001 statistical significance. *CDF* cumulative distribution function, *MRDEGs* macrophage-related differentially expressed genes.

To explore the expression differences of MRDEGs in COADREAD patient samples in TCGA-COADREAD dataset, R package "ConsensusClusterPlus" was employed to identify different subtypes of COADREAD disease related to COADREAD in TCGA-COADREAD dataset on the basis of expression levels of 13 MRDEGs (ADAM8, C5AR1, CCL22, CIITA, CTSD, CXCL8, CXCL9, FABP4, MMP3, MS4A1, SPP1, TIMP1, WNT5A) using the consistency clustering method. Finally, two COADREAD disease subtypes (cluster1 and cluster2) were identified (Fig. 10A). COADREAD disease subtype 1 (cluster1) contained 360 samples and COADREAD disease subtype 2 (cluster2) contained 284 samples. PCA was implemented on the expression data matrix of two subtypes of COADREAD disease samples in TCGA-COADREAD dataset. It demonstrated notable dissimilarities between the two COADREAD disease subtypes based on their expression matrices (Fig. 10B). We also showed the Delta plot (Fig. 10C) and cumulative distribution function (CDF) plot (Fig. 10D) of different numbers of clusters in the consistency clustering results and the consistency clustering CDF plot. The figure shows that the unsupervised clustering of the TCGA-COADREAD dataset is most consistent when using k = 2 as the number of clusters.

In addition, the variation in expression of 13 MRDEGs between two COADREAD disease subtypes (cluster1 and cluster2) in TCGA-COADREAD dataset was examined utilizing Mann–Whitney *U* test, and a group comparison graph was employed to present the results (Fig. 10E). The group comparison graph reveals significant variations in expression of 13 MRDEGs between cluster1 and cluster2 in TCGA-COADREAD dataset (P < 0.001).

Then we plotted the ROC curves of 13 MRDEGs (ADAM8, C5AR1, CCL22, CIITA, CTSD, CXCL8, CXCL9, FABP4, MMP3, MS4A1, SPP1, TIMP1, WNT5A) in the two COADREAD disease subtypes of TCGA-COADREAD dataset (Fig. S5).

### Mutation analysis of MRDEGs in CCRC patients

**Figure 11:**
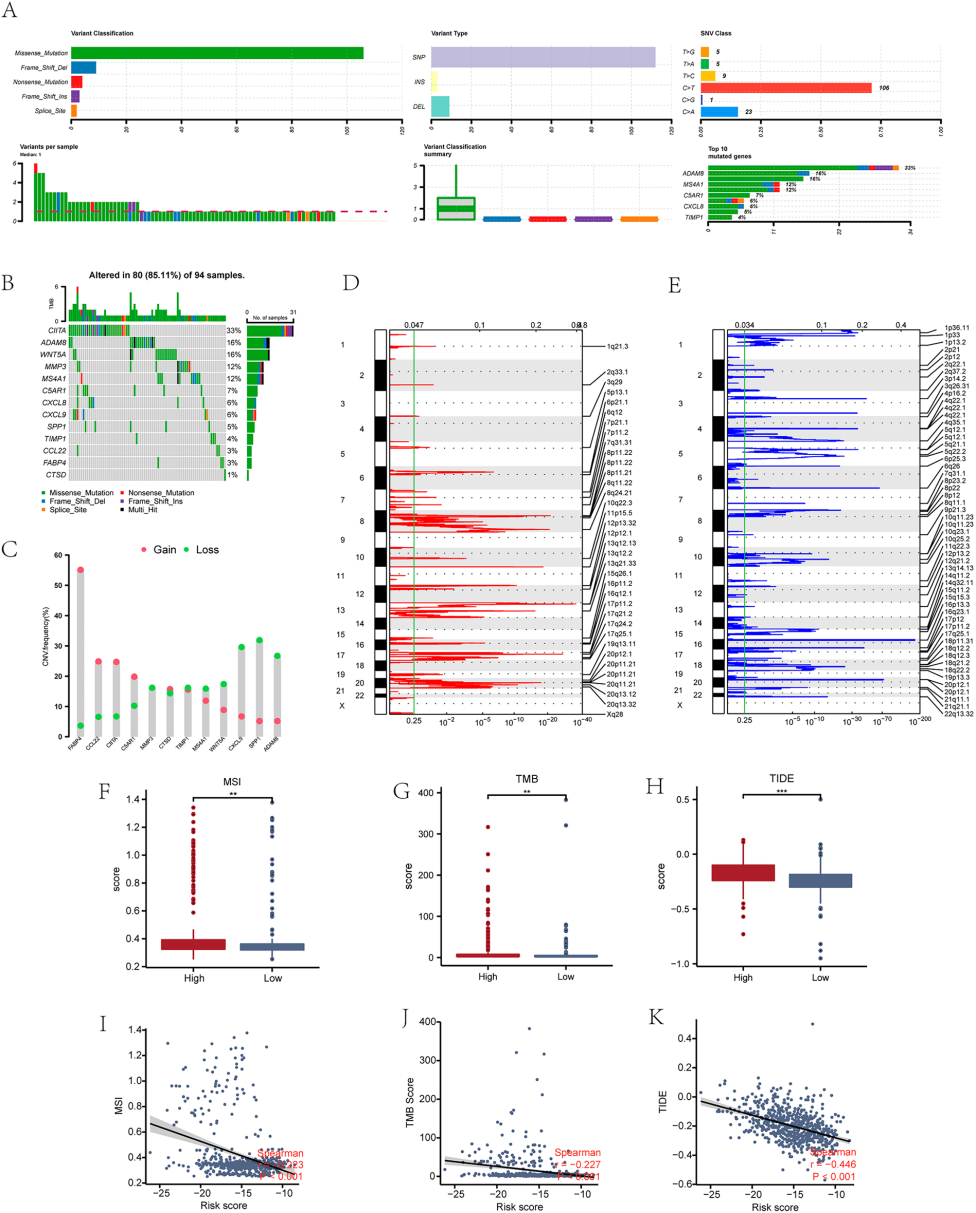
Mutation analysis of MRDEGs in COADREAD patients. (**A**,**B**) MRDEGs somatic mutation status (**A**) and proportion results (**B**) in COADREAD patients. (**C**) MRDEGs copy number variation in COADREAD patients. (**D-E**) Genes with increased (**D**) and decreased (**E**) copy numbers in COADREAD patients. (**F–H**) Group comparison charts of microsatellite instability (MSI) (F), tumor mutation burden (TMB) (**G**), and tumor immune dysfunction and exclusion (TIDE) scores (**H**) in COADREAD groups with high/low risks. (**I-K**) Scatter plots of correlation between MSI (**I**), TMB (**J**), TIDE score (**K**) and risk score. **P < 0.01 refers to high significant difference, ***P < 0.001 to extremely high significant difference. Absolute values of 0.3–0.5 refer to weak correlation, while values below 0.3 indicate refer to no correlation.

To analyze the mutation status of 13 MRDEGs (ADAM8, C5AR1, CCL22, CIITA, CTSD, CXCL8, CXCL9, FABP4, MMP3, MS4A1, SPP1, TIMP1, WNT5A) in COADREAD patients in TCGA-COADREAD dataset, mutation of 13 MRDEGs from COADREAD patient samples in TCGA-COADREAD dataset were analyzed and visualized utilizing R package maftools. The analysis revealed the presence of five main types of somatic mutations in the body cells: Missense Mutation, Frame Shift Deletion, Nonsense Mutation, Frame Shift Insertion, and Splice Site mutation. Missense mutations accounted for most of them (Fig. 11A). Most of the mutations observed in the 13 MRDEGs in COADREAD patients were SNPs, with a small number of insertions (INS) and deletions (DEL) also detected. Furthermore, the most frequent single nucleotide variant (SNV) observed in COADREAD patients was the C > T transition, followed by C > A (Fig. 11A). Then we showed all the somatic mutations of 13 MRDEGs in COADREAD patients (Fig. 11B).

We conducted an analysis on the CNV of 13 MRDEGs in TCGA-COADREAD dataset of COADREAD patients. We downloaded and merged the CNV data of COADREAD patients and analyzed it using GISTIC 2.0 and visualized the results (Fig. 11C–E). The results indicated a high frequency of amplifications and deletions of 13 MRDEGs in COADREAD patient samples, among which FABP4, CCL22, CIITA and other genes had higher amplification frequencies while CXCL9, SPP1 and ADAM8 had higher deletion frequencies (Fig. 11C).

We analyzed MSI and TMB data, as well as TIDE algorithm evaluation TIDE score data for COADREAD patients in TCGA-COADREAD dataset. Then we created grouping comparison graphs (Fig. 11F–H) and correlation scatter plots (Fig. 11I–K) to compare the patients’ risk scores. The results showed that MSI, TMB, and TIDE scores had statistically marked differences between patients with high/low risks (P < 0.05). Higher TIDE scores denote higher possibility of tumor immune escape in patients with high risk in contrast to those with low risk. The correlation scatter plot results showed a weak linear correlation between MSI data, TMB data, TIDE scores evaluated by TIDE algorithm, and risk scores.

### Immune infiltration analysis of CRC (CIBERSORT)

**Figure 12:**
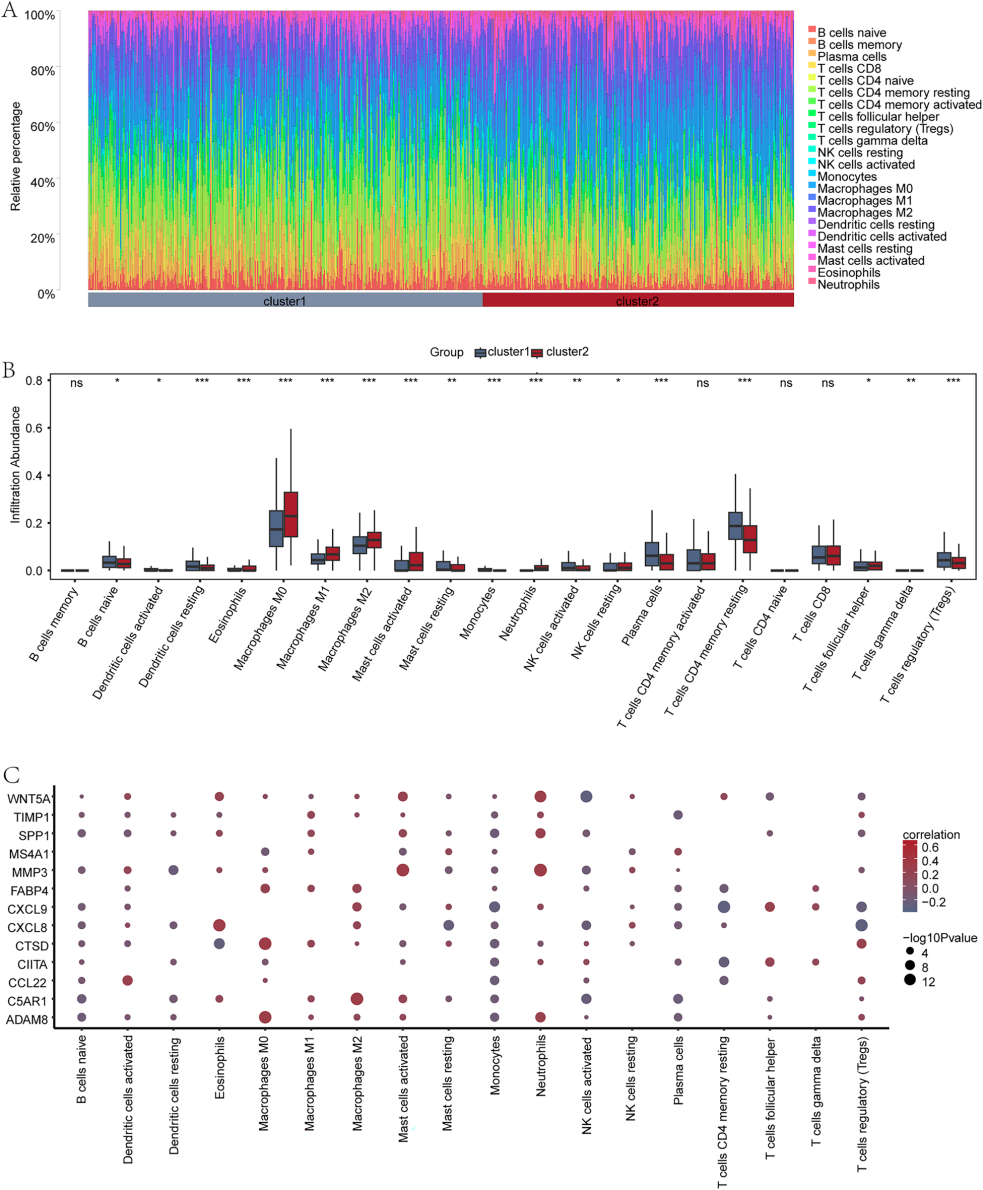
Immune infiltration analysis of CRC group (CIBERSORT). (**A**) The bar chart displays immune cell infiltration results of 22 immune cells in two groups (cluster1 and cluster2) of CRC patient. (**B**) The group comparison chart illustrates differences in the abundance of immune cell infiltration in two groups (cluster1 and cluster2) of CRC patients. (**C**) The heatmap shows correlation analysis results of MRDEGs and immune cells expressed between different groups (cluster1 and cluster2) in CRC patients. The symbol ns indicates no statistical significance (p > 0.05), *P < 0.05 refers to significant difference, **P < 0.01 to high significant difference, and ***P < 0.001 to extremely high significant difference. MRDEGs refer to Macrophage-related differentially expressed genes. In the correlation heatmap, a red circle denotes a positive correlation of the gene with abundance of immune cell infiltration, with larger circle representing stronger correlation. A blue circle represents a negative correlation of the gene with abundance of immune cell infiltration, with larger circle representing stronger correlation.

The correlation between the expression profiles of 22 immune cells in different groups (cluster1 and cluster2) in colon cancer patients were analyzed utilizing CIBERSORT algorithm. On the basis of immune infiltration analysis results, a bar chart (Fig. 12A) was generated to display the infiltration status of these 22 immune cells in each sample of colon cancer patients.

Differential expression of 22 immune cells in two groups (cluster1 and cluster2) in CRC patients was analyzed (Fig. 12B). The analysis revealed extremely significant differences of 11 immune cells, including dendritic cells resting, eosinophils, macrophages M0, M1, M2, mast cells activated, monocytes, neutrophils, plasma cells, T cells CD4 memory resting, and T cells regulatory (Tregs) in expression levels between the two groups (P < 0.001). Three immune cells (T cells gamma delta, NK cells activated, mast cells resting) had significant differences (P < 0.01), and four immune cells (B cells naive, NK cells resting, dendritic cells activated, T cells follicular helper) showed certain differences (P < 0.05).

We showed correlation heat map (Fig. 12C) of 13 MRDEGs (ADAM8, C5AR1, CCL22, CIITA, CTSD, CXCL8, CXCL9, FABP4, MMP3, MS4A1, SPP1, TIMP1, WNT5A) with statistically significant immune cell infiltration abundance (P < 0.05). There was a strong correlation between infiltration abundance of Neutrophils and MMP3 among MRDEGs in different groups (cluster1 and cluster2) of colon cancer patients. In the COADREAD subtype, the differential expression of M1 and M2 can be interpreted from multiple perspectives. Firstly, colorectal cancer, characterized by high heterogeneity, exhibits distinct molecular subtypes. M1 and M2 macrophages represent two different activation states, possibly influenced by diverse immune environments and cell signal regulations specific to these subtypes, resulting in their expression differences across various subtypes. Secondly, different subtypes may display varying levels of inflammation, with M1 macrophages commonly associated with inflammation. Therefore, in subtypes with more pronounced inflammation, the expression of M1 may be more prominent. Further exploration is relevant to the immunological characteristics and clinical prognosis of colorectal cancer. Immune cell infiltration positively correlates with anti-tumor immune responses, and the heightened expression of M1 macrophages may reflect a stronger anti-tumor immune response, associated with better prognosis, aligning with previous research findings. However, significant expression of M2 in certain subtypes may indicate immune suppression and tumor escape, consistent with the general notion that groups exhibiting M2 characteristics tend to have poorer prognoses.

Finally, the expression differences between M1 and M2 may have potential biological implications for patient prognosis. High expression of M1 may serve as an independent predictor for a better prognosis in colorectal cancer patients, as its robust anti-tumor immune response helps restrict tumor growth and spread. Conversely, elevated expression of M2 may suggest immune escape and tumor progression, correlating with adverse prognosis, possibly reflecting an immune-suppressive microenvironment conducive to tumor escape and growth. These findings provide crucial insights into understanding the functional disparities of M1 and M2 in colorectal cancer subtypes and offer valuable information for potential therapeutic strategies.

### Clinical correlation analysis of prognostic MRDEGs

We investigated whether the expression levels of 13 prognostic MRDEGs were related to clinical features in COADREAD patients. The correlation of high and low expressions of these MRDEGs with different clinical pathological characteristics was examined (Fig. S6).

### In vitro and vivo analyses

**Figure 13:**
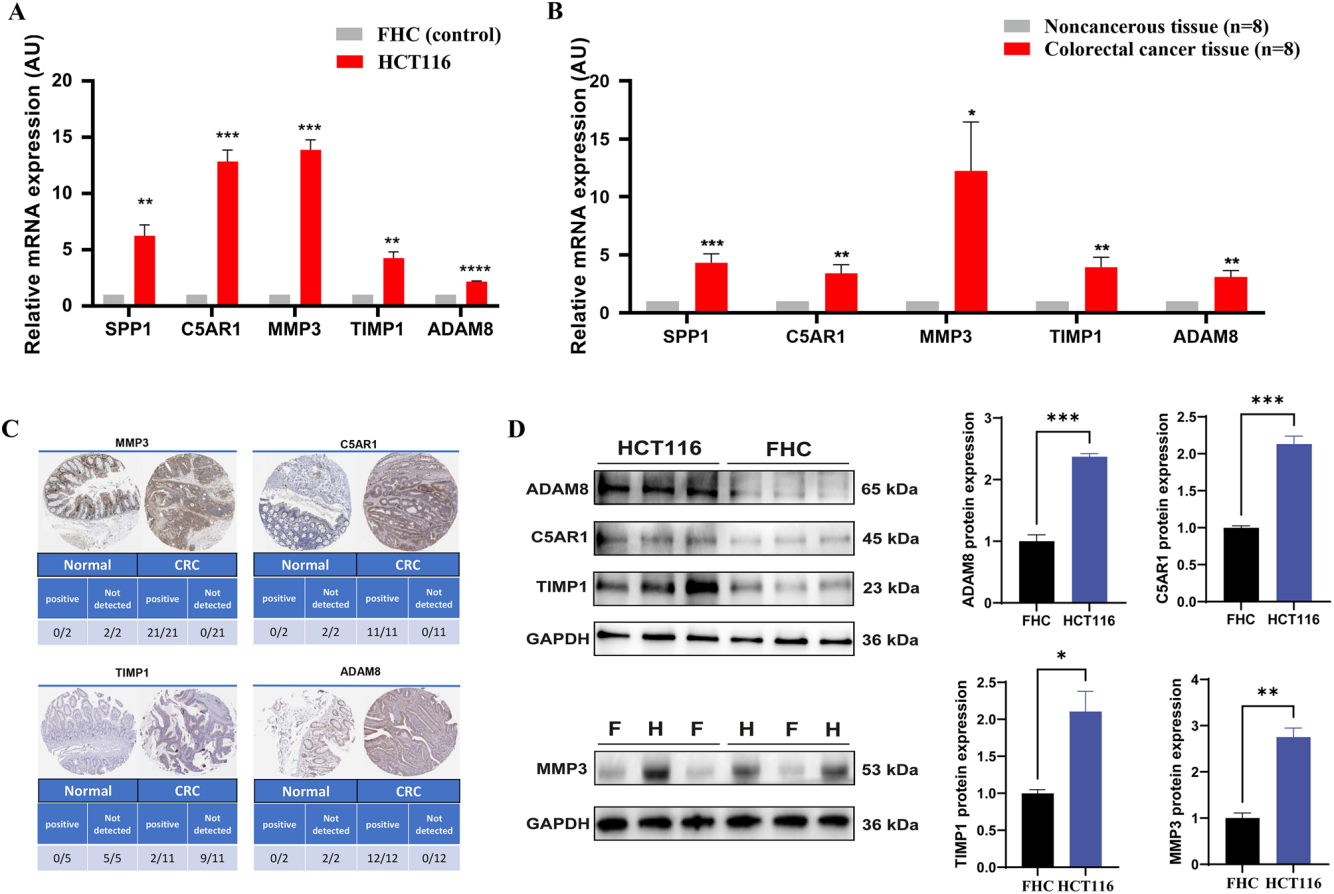
The mRNA levels of hub genes. (**A**) The gene expression levels of hub genes in HCT116 and FHC cell. The FHC cells are normal human intestinal epithelial cells, while HCT116 cells are human colorectal carcinoma cells. (**B**) The gene expression levels of hub genes in CRC tissue and Noncancerous tissue from human patients. (**C**) Representative images and statistics of IHC staining for MMP3, C5AR1, TIMP1, ADAM8 in colon tissues and CRC from the Human Protein Atlas dataset. (**D**) The protein expression levels of MMP3 ("F" represents FHC cells, and "H" represents HCT116 cells), ADAM8, TIMP1, and C5AR1 were assessed in FHC cells and HCT116 cells, with GAPDH serving as a reference. Statistical significance levels were denoted as *P < 0.05, **P < 0.01, ***P < 0.001, and ****P < 0.0001.

Real-time quantitative reverse transcription PCR was employed to detect the mRNA expression levels of hub genes in the HCT116 colorectal cancer cell line, normal colon epithelial cells, eight colorectal cancer (CRC) patients, and eight control subjects in adjacent tissues. This validation aimed to assess the reliability of the hub genes. The results demonstrated a significant upregulation of SPP1, C5AR1, MMP3, TIMP1, and ADAM8 expression in HCT116 cells compared to normal colon epithelial cells (Fig. 13A). Consistently, in the clinical samples, the expression levels of SPP1, C5AR1, MMP3, TIMP1, and ADAM8 were significantly higher in CRC patients compared to the control tissues (Fig. 13B), corroborating the aforementioned findings. The protein expression of SPP1, C5AR1, MMP3, TIMP1, and ADAM8 was examined using the Human Protein Atlas database from CRC patients, revealing a similar trend for C5AR1, MMP3, TIMP1, and ADAM8 (Fig. 13C). Additionally, we observed a significant increase in protein expression levels of MMP3, TIMP1, ADAM8, and C5AR1 in HCT116 cells compared to FHC cells, consistent with the mRNA expression results. (Fig. 13D).

## Discussion

The crucial role of macrophages in tumor proliferation is increasingly recognized. Among the two classes of macrophages, M2 class macrophages are thought to depress immunity against tumor. Tumor associated macrophages are mostly thought to be similar with M2 macrophages. Approaches through targeting macrophages in TME are proposed to treat cancer^23^. In tumor immunotherapy, targeting macrophages has several advantages. Hypoinvasive is a main obstacle to T cell-based anti-cancer therapies, while in the TME, macrophages make up 30–50% of infiltrating immune cells. Macrophage infiltration in tumors is mainly derived from circulating monocytes, and macrophage-based therapeutic strategies are easily to employ in the clinic due to the availability of peripheral blood mononuclear cells. In order to do so, it is important to understand what specific changes are happening in the TAM genes.

In our study, we obtained the Macrophage scores by performing ssGSEA on the TCGA-COADREAD dataset to score the MRGs. The samples were categorized into groups with high/low scores by utilizing median phenotype score, and DEGs was subsequently conducted. Then DEGs were intersected with MRGs to obtain the MRDEGs, and GO-KEGG enrichment analyses were implemented on MRDEGs, as well as GSEA on TCGA-COADREAD dataset. We identified key genes (ADAM8, C5AR1, CCL22, CIITA, CTSD, CXCL8, CXCL9, FABP4, MMP3, MS4A1, SPP1, TIMP1, WNT5A) by performing LASSO model selection on the genes obtained from the intersection of MRDEGs and co-expression module-related genes, and subsequently performed consistent clustering analysis, Cox analysis, immune infiltration analysis, mutation analysis, clinical correlation analysis, and we conducted differential expression analysis of critical genes within GEO dataset. Despite the publication of the dataset, previous research has not emphasized the connection between macrophage infiltration and CRC in gene expression analysis. Furthermore, the precise function of TAMs in CRC has not been definitively established. Thus, this study aims to broaden the screening parameters for CRC through bioinformatics analysis, improve the sensitivity of CRC diagnosis standards, and identify potential macrophage-related genes in CRC.

Prior research has indicated that MMPs are primarily expressed by macrophages, and are involved in regulating the equilibrium between deposition and degradation of the extracellular matrix^24^. MMP3 is a family member of zinc-dependent endopeptidases. It is mostly secreted by immune cells (i.e. neutrophils, mononuclear macrophages), endothelial cells, and cancer cells. MMP3 has been illustrated to have a vital part in extracellular matrix degradation^25,26^, and both MMP3 and TIMP1 have been utilized as biomarkers for CRC^27,28^. In addition, the level of MMP3 in the serum has a direct association with disease activity, with elevated MMP3 levels leading to an increase in disease activity. There is evidence suggesting that TAMs may engage with the complement system to facilitate tissue remodeling^29^. Nonetheless, the activity of matrix metalloproteinases (MMPs) can be neutralized by TIMP1. We speculate that high expression of MMP3 in CRC patients leads to an upregulation of TIMP1. According to a previous report, TIMP1 serves as a prognostic marker for colon cancer development and metastasis via the MAPK and AKT-pi3k/AKT pathways^30^. This finding aligns with our bioinformatics analysis and validation results. The association between TAMs and the upregulation of MMP3 and TIMP1 in CRC progression needs further exploration. A disintegrin and metalloprotease domain 8 (ADAM8) belongs to a human ADAM family, containing disintegrin and metalloproteinase domains^31^. ADAM proteins participate in different cellular processes, comprising protein hydrolysis, cell fusion, migration, adhesion, membrane shedding, etc.^32,33^. ADAM8 can activate metalloproteinases, leading to the promotion of matrix remodeling. Research has demonstrated that inhibiting activities of ADAM8 and MMP can impede invasive and migratory abilities of drug-resistant colon cancer cells^34^. ADAM8 has also been suggested as an underlying biomarker for CRC^35^. Our analysis suggests that ADAM8 is a potential macrophage-related biomarker for CRC, and its mechanism of function requires further investigation.

The SPP1 gene encodes a protein associated with osteoclasts attachment to mineralized bone matrix, and also functions as a cytokine that increases the expression of interferon-γ and interleukin-12. A subtype of TAMs, called SPP1^+^ macrophages, have been reported to exhibit unique characteristics and have immunosuppressive properties. These macrophages are positively correlated with markers of epithelial-mesenchymal transition, a process related to increased tumor growth and metastasis. SPP1^+^ TAMs mainly interact with fibroblasts and promote angiogenesis and tumor metastasis^36^ which is mediated by cytokines encoded by IL1A, IL1B, or TGFB1. The promotion of stromal TME through ECM remodeling facilitates tumor growth and invasion, exacerbating CRC progression^37^. Targeting SPP1^+^ macrophages may be a possible strategy for anti-tumor growth and metastasis. The discovery suggests that increased levels of SPP1 in macrophages surrounding tumors are linked to unfavorable outcomes in patients with CRC. SPP1^+^ macrophages exhibit significant promise in the field of CRC immunotherapy.

As a vital component of the immune response, the complement system is able to react swiftly and comprehensively to both external microbial threats and internal challenges. Made up of a range of plasma and membrane proteins, this system plays a critical role in upholding immune homeostasis while simultaneously facilitating immune surveillance^38^. The complement system not only functions in the extracellular environment, but also inside cells. How activation of the complementary system functions in tumor cells remain unknown. In TME, C5a recruits immune suppressive cells expressing the C5AR1 receptor, and high C5aR1 levels correlate with a poor prognosis in CTSD^39^. Our findings indicate that MRDEGs are significantly enriched in functions related to chemotactic factors, such as cytokine activity, chemokine receptor binding, receptor-ligand activity and chemotactic factor signaling pathways. Complement 5a (C5a) is a cell cytokine-like peptide produced during the complement system activation process, and there is literature suggesting that C5a stimulates macrophage polarization and promotes colon cancer metastasis^40^. Despite extensive research on intracellular complement activation in various cell types, little attention has been paid to its role in tumor cells. However, recent animal studies have demonstrated that knocking out C5AR1 inhibits β-catenin expression and activation in intestinal tissue, resulting in a significant decrease in CRC development. The findings indicate that C5AR1 could be taken as a underlying therapeutic target for CRC^41^. Investigating the intricate mechanisms of complement system and macrophage interaction in CRC would be a fascinating area for future research.

**Figure 14:**
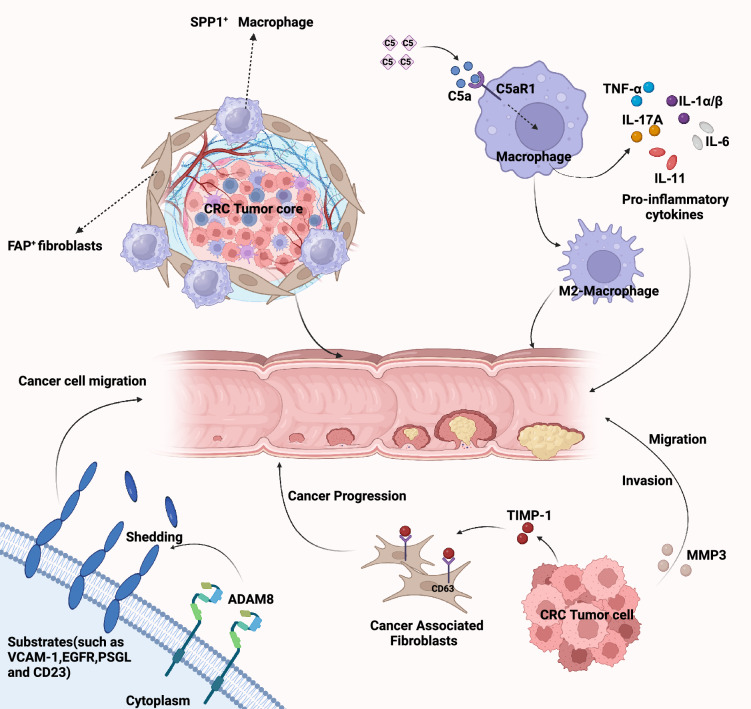
Illustration of the roles of SPP1, C5AR1, MMP3, TIMP1, and ADAM8 in the progression of colorectal cancer. Created by biorender.

In our study, we utilized WGCNA and LASSO screening methods, and verified via real-time quantitative PCR, to ultimately identify SPP1, C5AR1, MMP3, TIMP1, ADAM8 as potential macrophage-related biomarkers for CRC^37,41–44^ (Fig. 14). The work mentioned provides new and valuable information about the key genes and underlying mechanisms of TAMs in CRC development. Studying these important genes in more detail can improve our understanding of how CRC progresses and assist us to recognize potential targets for treatment. However, this study still presents areas that require further investigation. Comparative analyses with other clinical subtypes and more in-depth functional analyses would be both intriguing and crucial. We intend to explore these directions in our future research endeavors.

## Conclusions

In conclusion, our study successfully identified five hub genes associated with macrophages, which could potentially collaborate in promoting CRC formation. It may even hold the promise of improving therapeutic approaches for colon cancer patients in clinical practice.

### Supplementary Information

Supplementary Figures.

Supplementary Table S1.

Supplementary Table S2.

## Data Availability

The original data for this study were obtained from TCGA database (https://portal.gdc.cancer.gov). All data generated or analysed during this study are included in this published article and its supplementary information files. Further inquiries can be directed to the corresponding authors.
