# Supplementary material for: Identification of a novel macrophage-related prognostic signature in colorectal cancer
Source: Sci Rep. 2024 Feb 2;14:2767. doi: 10.1038/s41598-024-53207-9 (PMC10837438; doi:10.1038/s41598-024-53207-9)
Supplement: Supplementary file 1 — Supplementary Figures. [file 41598_2024_53207_MOESM1_ESM.pdf]

Supplementary Information

Identification of a Novel Macrophage-Related Prognostic Signature in Colorectal cancer

Dongfa Lin[1], *Tingjin Zheng*[2], *Shangyuan Huang*[3], *Rui Liu*[1],

Shuwen Guan [1,] * & *Zhishan Zhang* [2,] *

[1] *Key Laboratory for Molecular Enzymology and Engineering, The Ministry of Education, Jilin University, School of Life Sciences, 130012, Changchun, China;*

[2]*Department of Clinical Laboratory, Quanzhou First Hospital Affiliated to Fujian Medical University, No. 248 East Street, Quanzhou City, Fujian 362000, China;*

[3] *Laboratory of Molecular neurobiology, Sheng Yushou center of Cell Biology and Immunology, Department of Genetics and Developmental Biology, School of Life Sciences and Biotechnology, Shanghai Jiao Tong University, 800 Dongchuan Rd., 200240, Shanghai, China;*

---

* Corresponding author: *E-mail address*: guanshuwen@jlu.edu.cn ; zhishanzhang@139.com

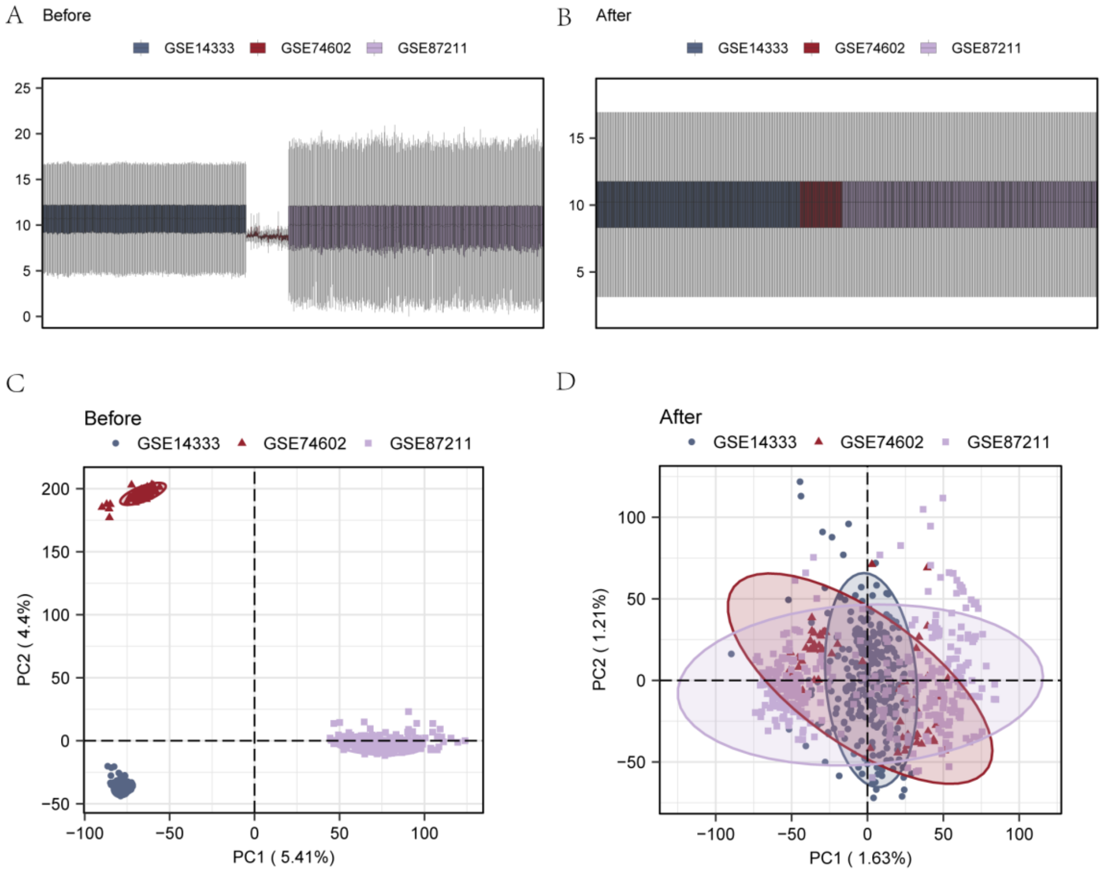

Figure S1 Box line plots and principal component analysis plots of sample distribution before and after GEO dataset merging. A-B. Box plots before (A) and after (B) merging GEO datasets. C-D. PCA plots before (C) and after (D) merging GEO datasets.

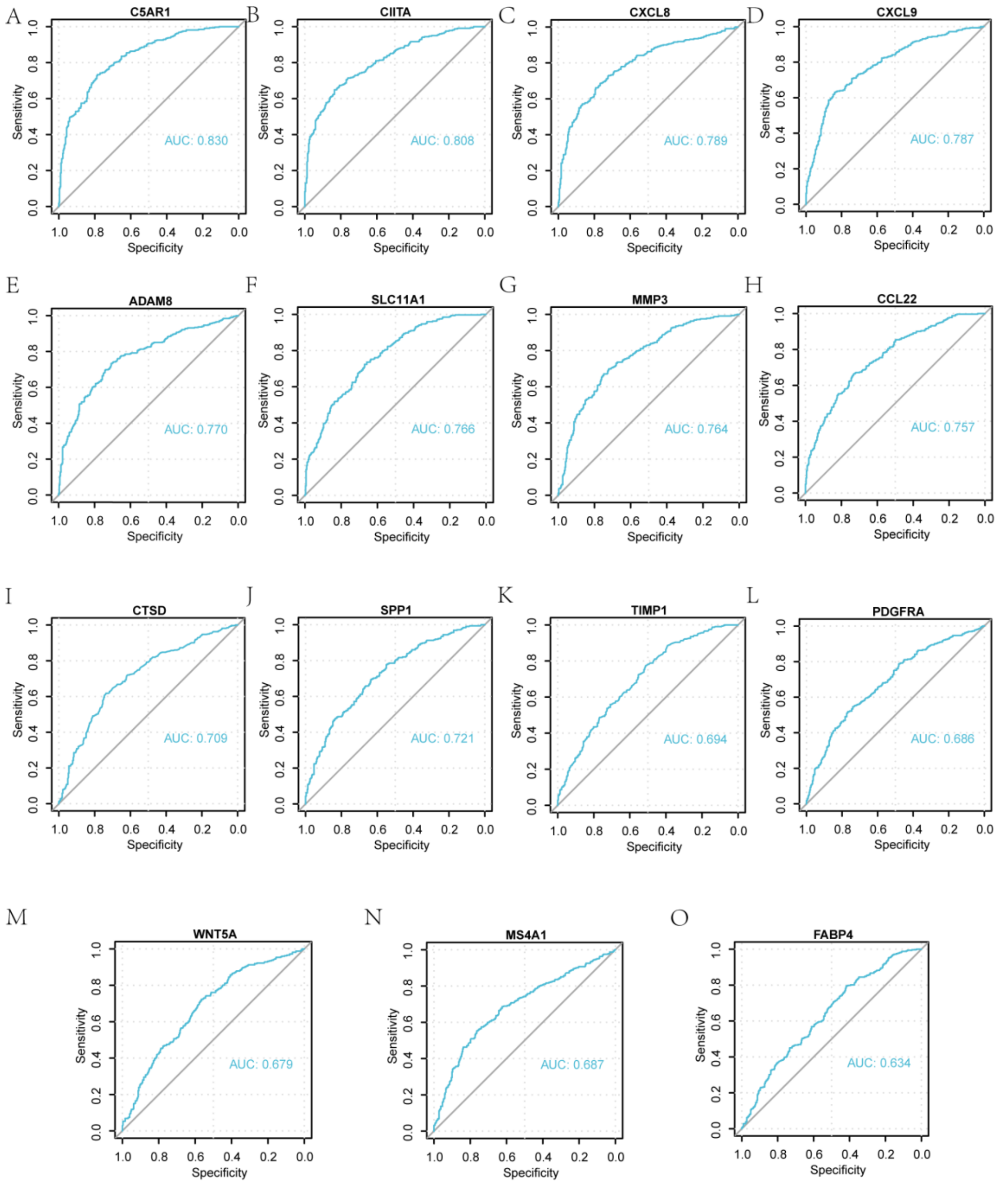

Figure S2 Expression of macrophage-related differentially expressed genes in the dataset TCGA-COADREAD. ROC curve analysis of DEGs C5AR1 (A), CIITA (B), CXCL8 (C), CXCL9 (D), ADAM8 (E), SLC11A1 (F), MMP3 (G), CCL22 (H), CTSD (I), SPP1 (J), TIMP1 (K), PDGFRA (L), WNT5A (M), MS4A1 (N), FABP4 (O) in TCGA-COADREAD dataset. When AUC of ROC curve is closer to 1, it indicates a better diagnostic effect. The AUC is considered to have high accuracy if it is between 0.7 and 0.9, whereas it has lower accuracy if it ranges between 0.5 and 0.7.

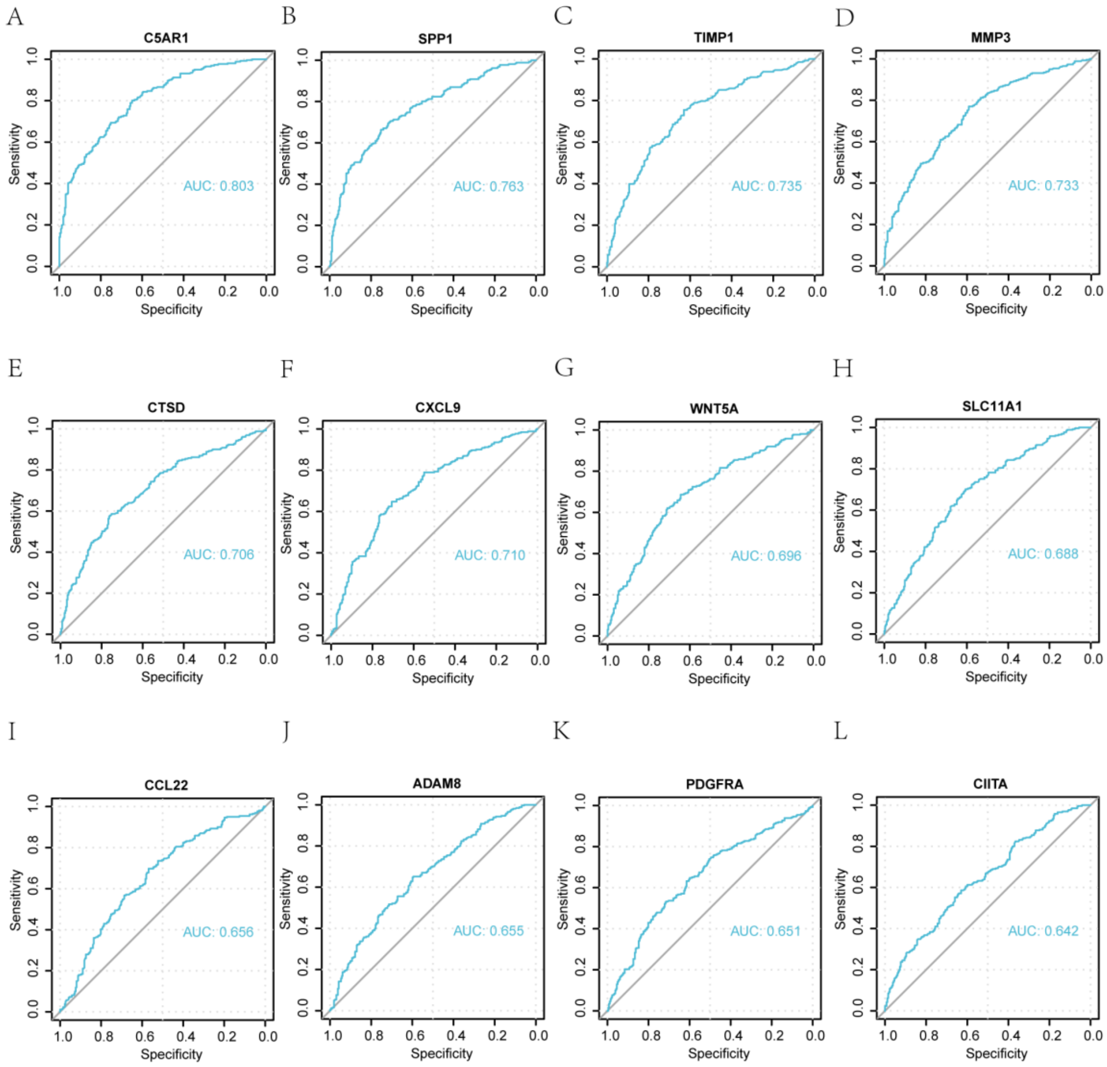

Figure S3 Expression of MRDEGs in GEO dataset. ROC curves of MRDEGs A-L C5AR1 (A), SPP1 (B), TIMP1 (C), MMP3 (D), CTSD (E), CXCL9 (F), WNT5A (G), SLC11A1 (H), CCL22 (I), ADAM8 (J), PDGFRA (K), CIITA (L) in GEO dataset. ROC curves with AUC closer to 1 indicate better diagnostic performance. AUC is more accurate when between 0.7 and 0.9, and less accurate when between 0.5 and 0.7.

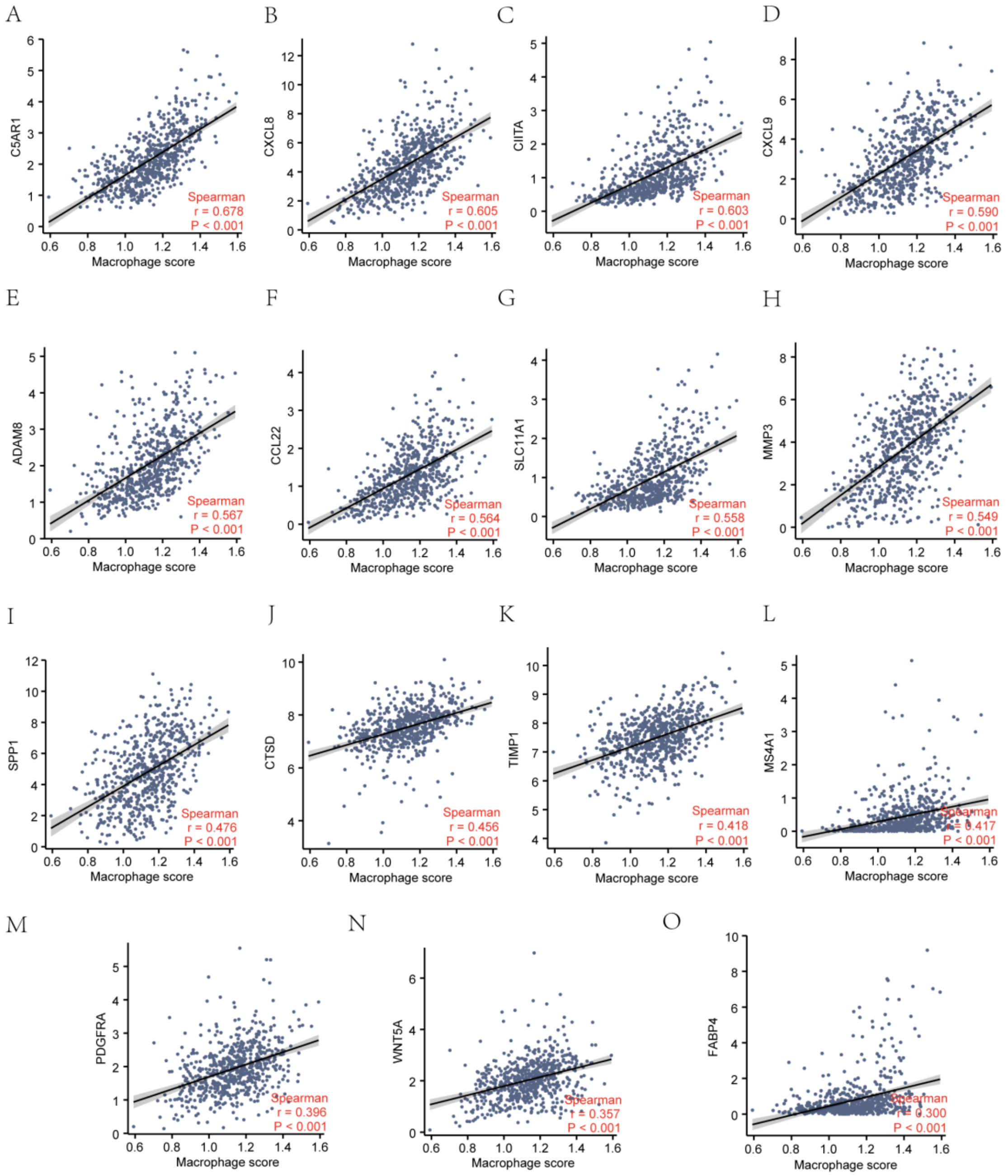

Figure S4 Correlation analysis between hub genes and macrophage scores. A-O. Hub genes and correlation analysis of macrophage score correlation scatter plot display. P<0.001, which means it has statistical significance. The absolute value of correlation coefficient (cor value) shows a weak correlation or no correlation below 0.3, a weak correlation between 0.3-0.5, a moderate correlation between 0.5-0.8, and a strong correlation above 0.8.

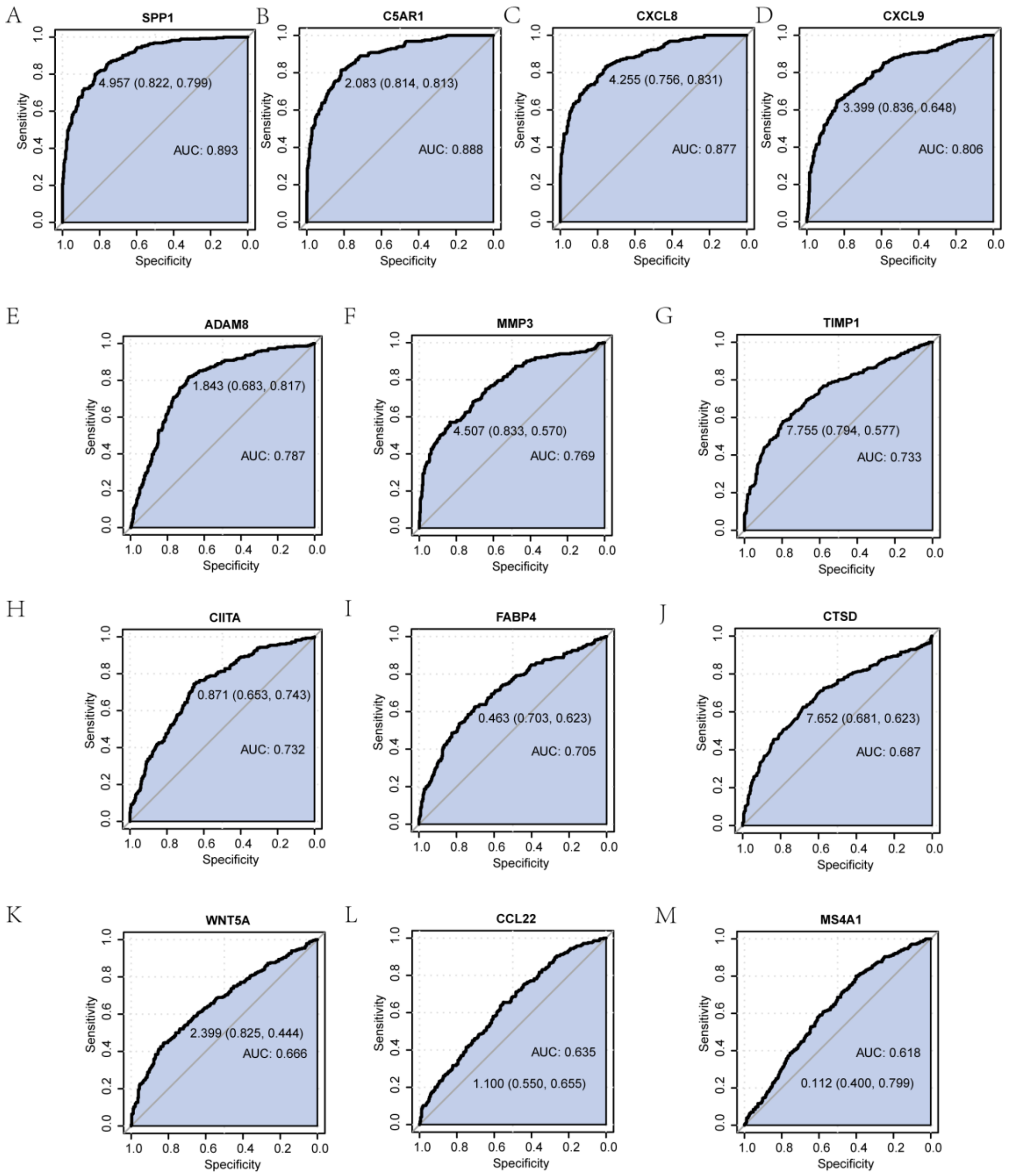

Figure S5 Construction of COADREAD associated disease subtypes. A-M. The ROC curve for SPP1 (A), C5AR1 (B), CXCL8 (C), CXCL9 (D), ADAM8 (E), MMP3 (F), TIMP1 (G), CIITA (H), FABP4 (I), CTSD (J), WNT5A (K), CCL22 (L), and MS4A1 (M) in different COADREAD disease subtypes is presented. The diagnostic effect is better when AUC in ROC curve is closer to 1. ROC: receiver operating characteristic curve. MRDEGs: Macrophage-related differentially expressed genes.

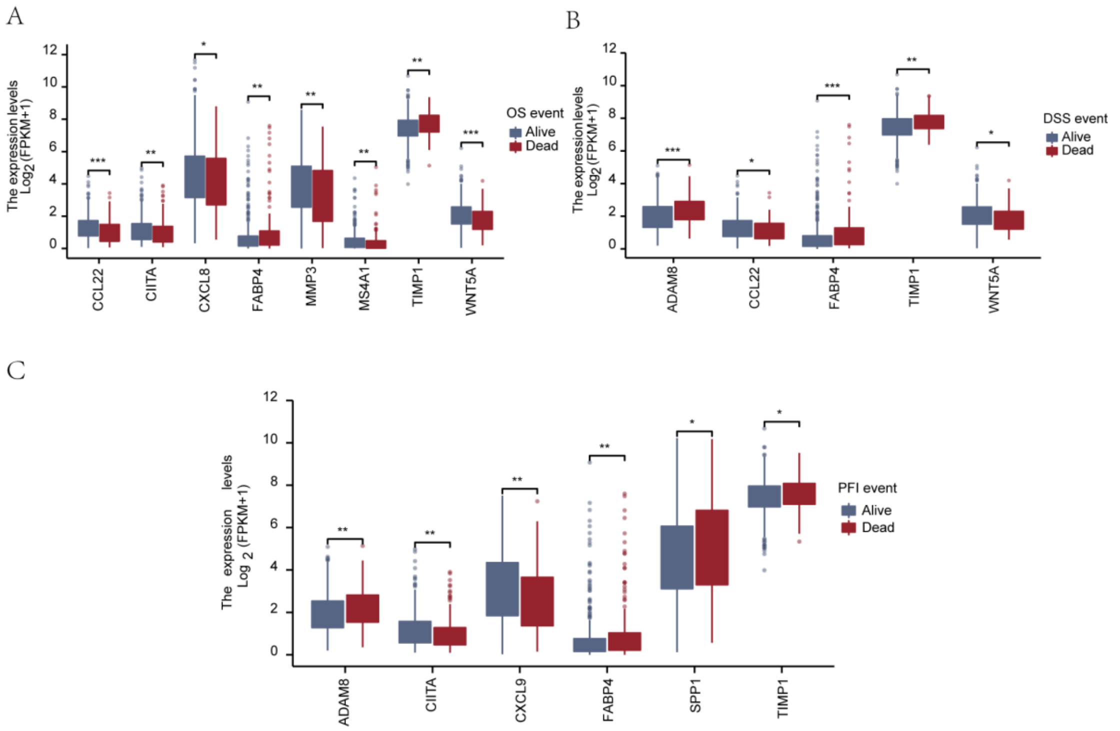

Figure S6 Clinical correlation analysis of MRDEGs. A-C. Correlation analysis of prognostic MRDEGs with overall survival (A), disease-specific survival (B), and progression-free interval (C). *P < 0.05 refers to significant difference, **P < 0.01 to high significant difference, ***P < 0.001 to extremely high significant difference. MRDEGs: Macrophage-related differentially expressed genes.

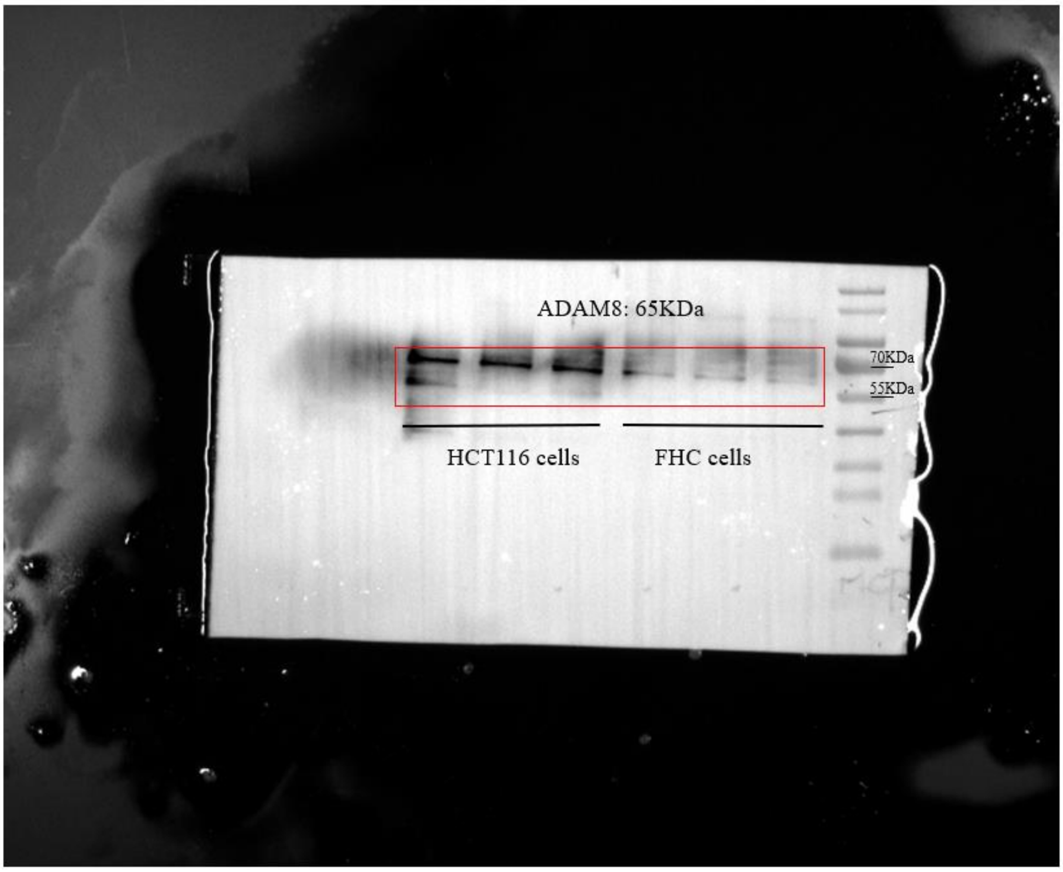
ADAM8: 65KDa
70KDa
55KDa
HCT116 cells
FHC cells

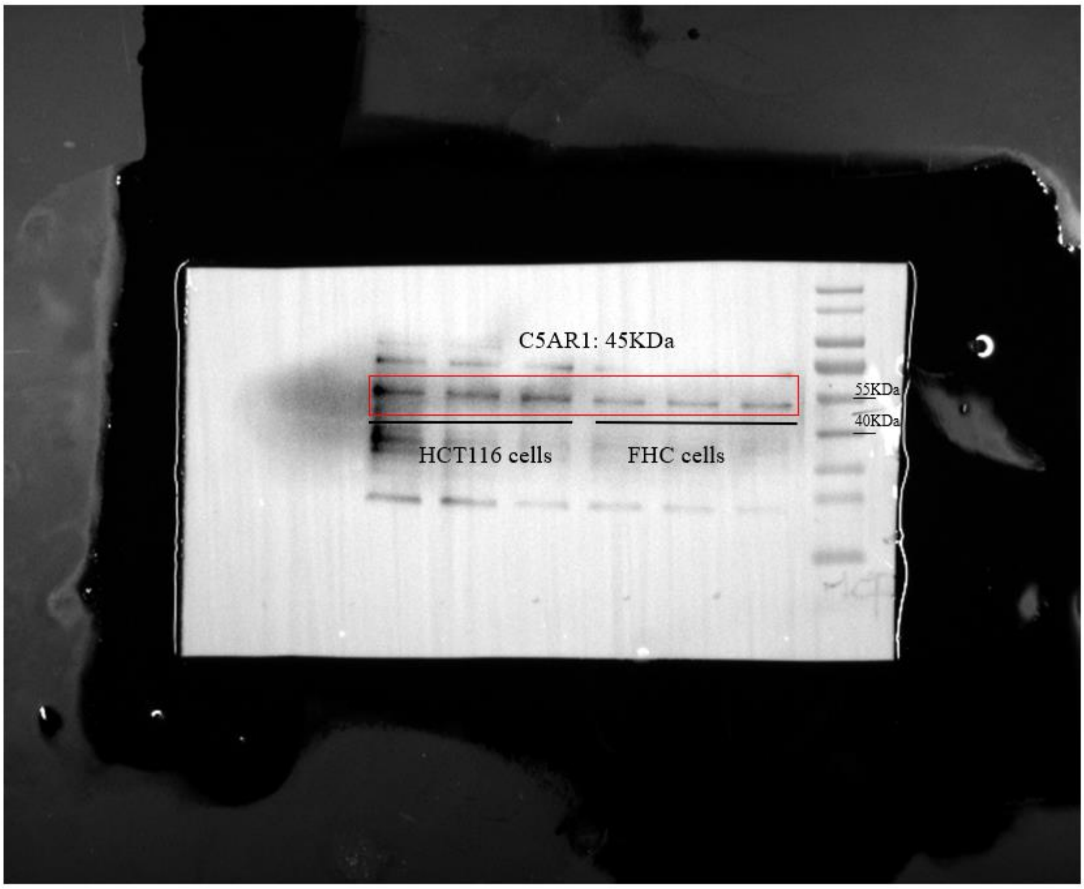
C5AR1: 45KDa
55KDa
40KDa
HCT116 cells
FHC cells

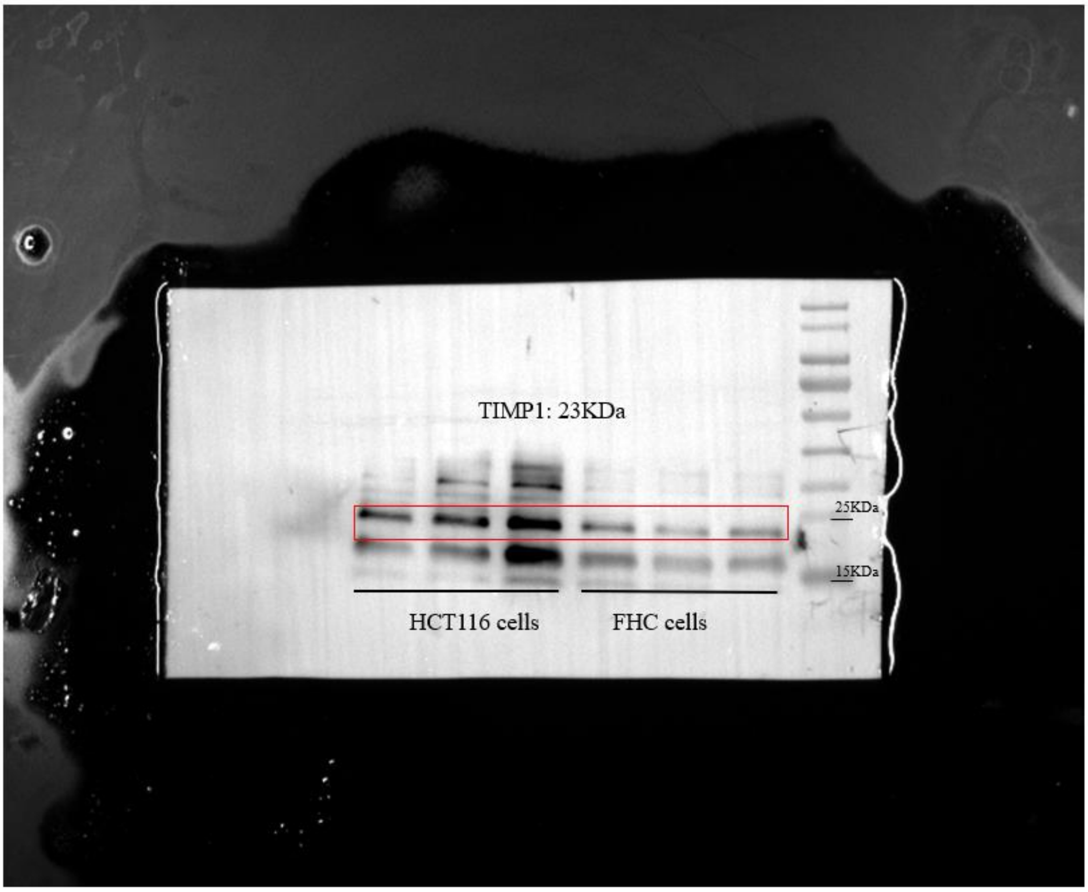
TIMP1: 23KDa
25KDa
15KDa
HCT116 cells
FHC cells

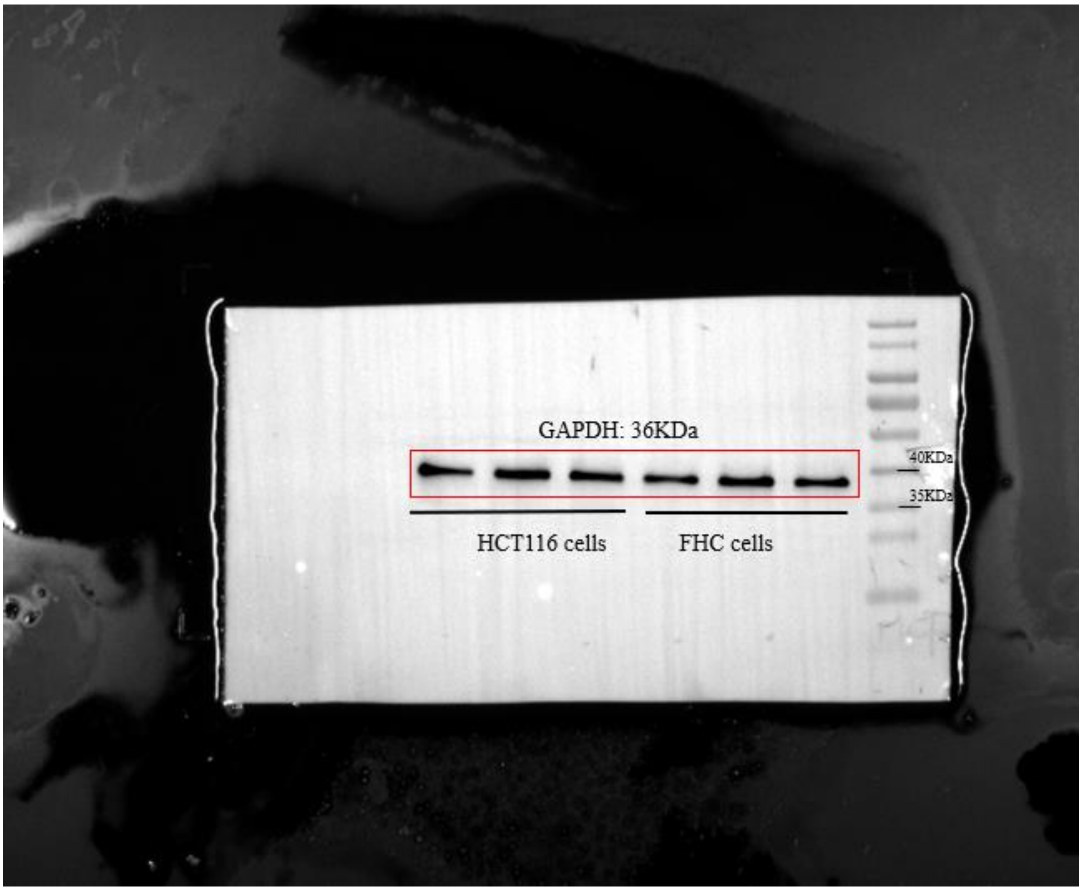
GAPDH: 36KDa
40KDa
35KDa
HCT116 cells
FHC cells

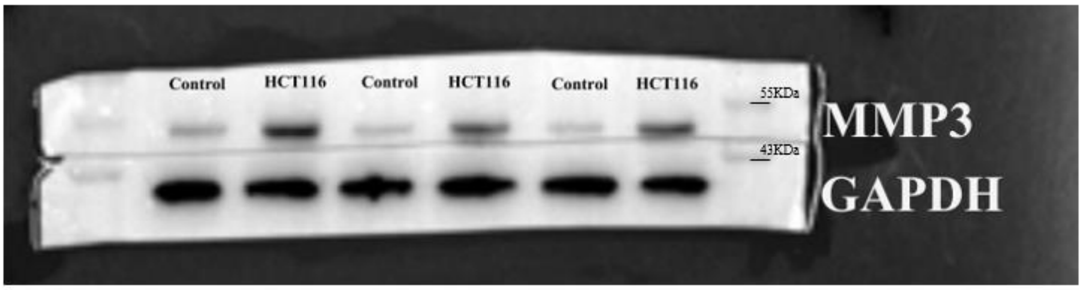
Control
HCT116
Control
HCT116
Control
HCT116
55KDa
43KDa
MMP3
GAPDH
